# Supplementary material for: A brainstem monosynaptic excitatory pathway that drives locomotor activities and sympathetic cardiovascular responses
Source: Nat Commun. 2022 Aug 29;13:5079. doi: 10.1038/s41467-022-32823-x (PMC9424289; doi:10.1038/s41467-022-32823-x)
Supplement: Supplementary file 1 — Supplementary Information [file 41467_2022_32823_MOESM1_ESM.pdf]

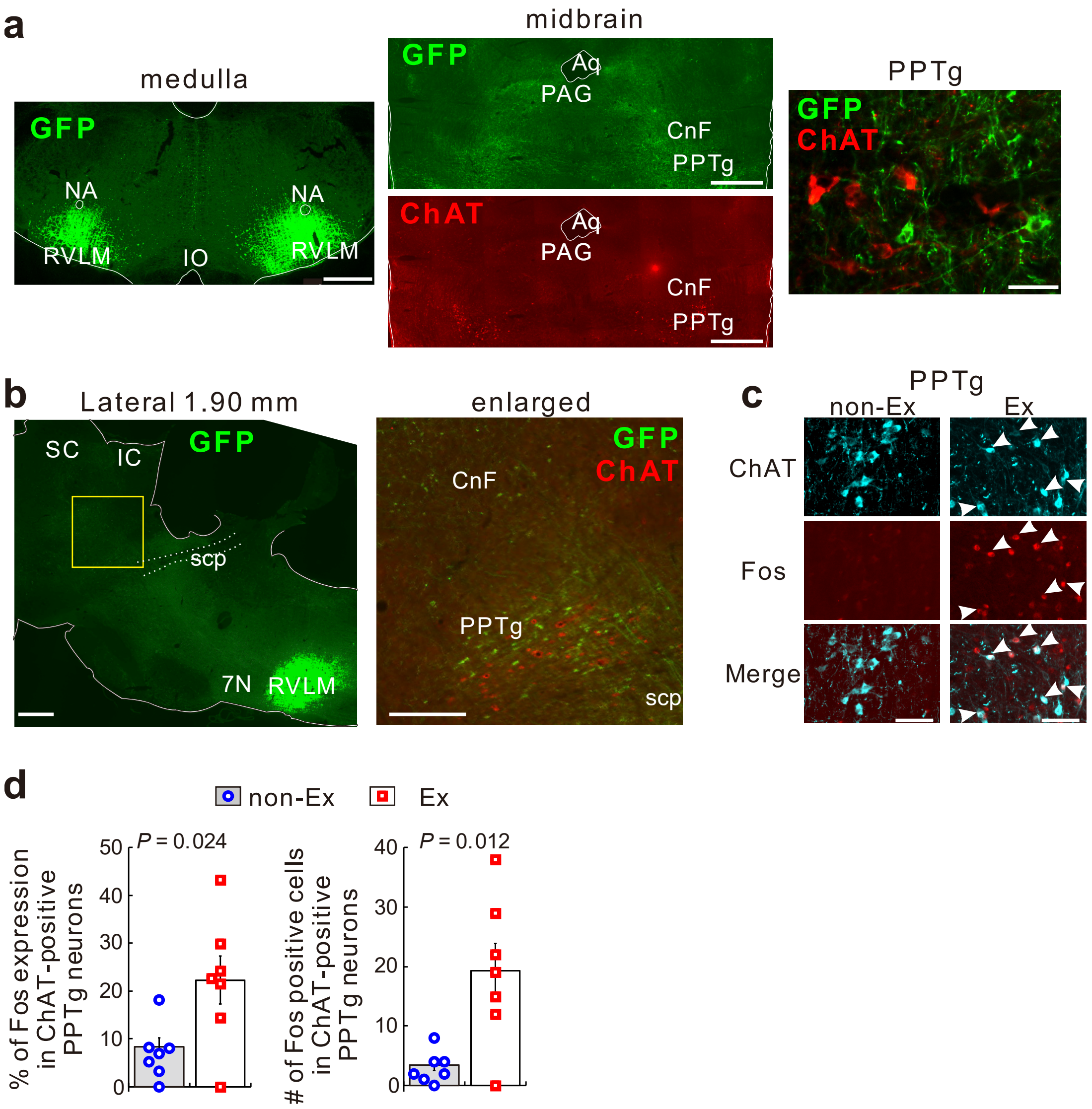

Supplementary Figure 1. GFP transduction in RVLM-projecting MLR neurons and Fos expression in MLR neurons. a GFP transduction (autofluorescence) in axonal fibers in the RVLM (left), and GFP- and ChAT-immunoreactive cell distribution in the midbrain (center) and in the PPTg (right). Scale bars: 1 mm (left, center) and 50 μm (right). **b** Sagittal section showing GFP transduction in the RVLM (left) and GFP- and ChAT-labeled neurons in the MLR (right). 7N: facial nucleus; IC: inferior colliculus; SC: superior colliculus, scp: superior cerebellar peduncle. Scale bars: 1 mm (left) and 500 μm (right). **c** Fos expression in ChAT-immunoreactive cells in the PPTg (arrowheads). Scale bar: 100 μm. d Comparisons of Fos-immunoreactive cells in ChAT-immunoreactive PPTg neurons between non-exercised controls and exercised rats (n = 7 for each group; all males). Data were analyzed by two-sided Welch's t-test; statistic information including t statistic values and degrees of freedom is presented in Supplementary Table 15. Data shown are means ± SEM. Source data are provided as a Source Data file.

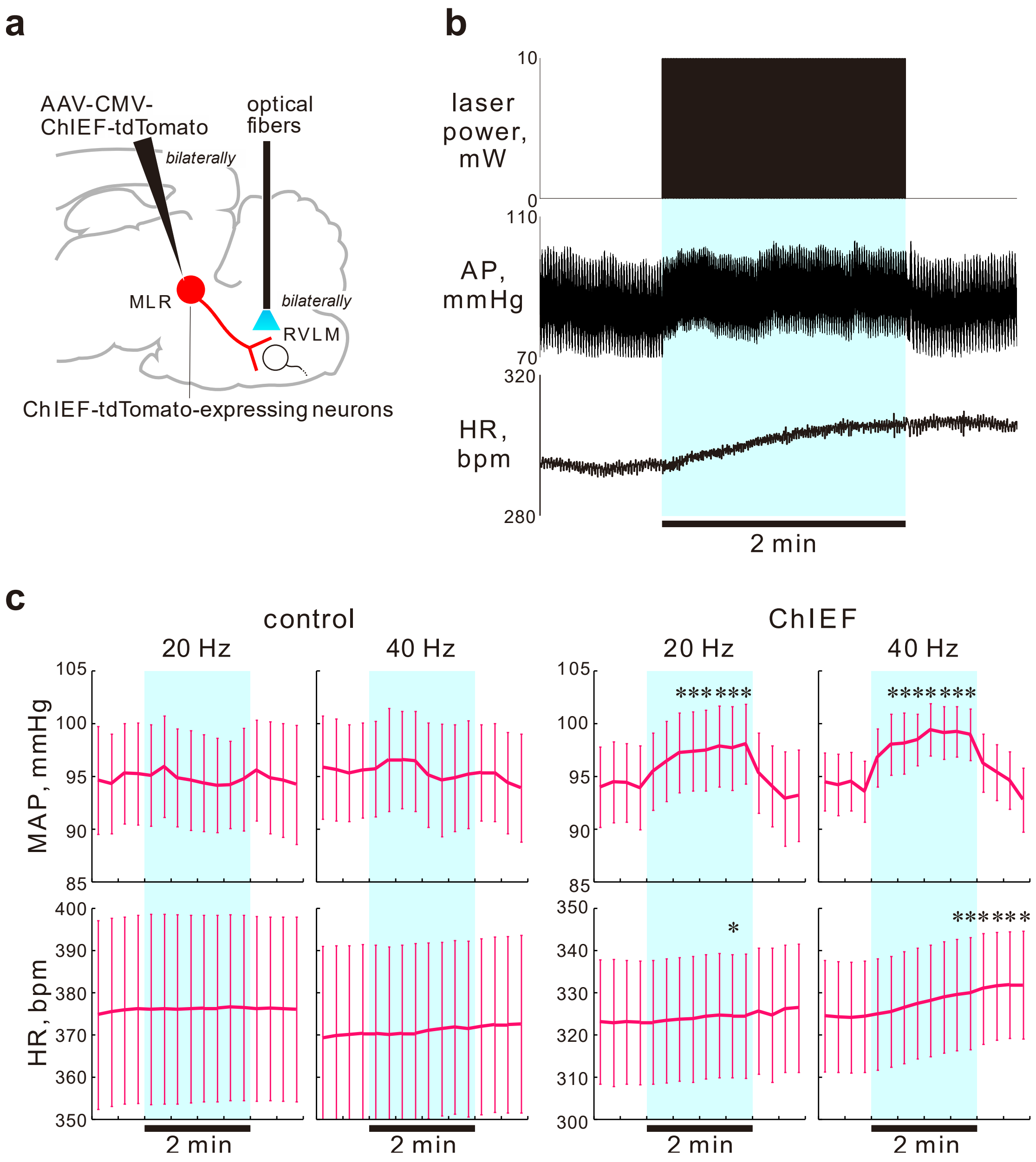

Supplementary Figure 2. AP and HR responses to optogenetic stimulation of MLR→RVLM neuronal axons. **a** Optogenetic stimulation of MLR-RVLM neuronal axons in anesthetized rats. **b** Representative recording during optogenetic stimulation (40 Hz) in a ChIEF-tdTomato-expressing anesthetized rat. **c** Time courses (15-s bins) in palGFP-expressing control (n = 4) and ChIEF-tdTomato-expressing male rats (n = 7). Data were analyzed via one-way RM ANOVA/Friedman one-way RM ANOVA by rank followed by Dunnett's *post hoc* test. Statistic information including statistic values and degrees of freedom is presented in Supplementary Table 15. *P < 0.05 vs. 60-s averaged baseline. Baseline values for c are reported in Supplementary Table 1. Data shown are means ± SEM. Source data are provided as a Source Data file. The brain section image used in the figure (**a**) was adapted from "Paxinos G & Watson C. The rat brain in stereotaxic coordinates. 6th edn. (Amsterdam, Academic Press/Elsevier, 2007)".

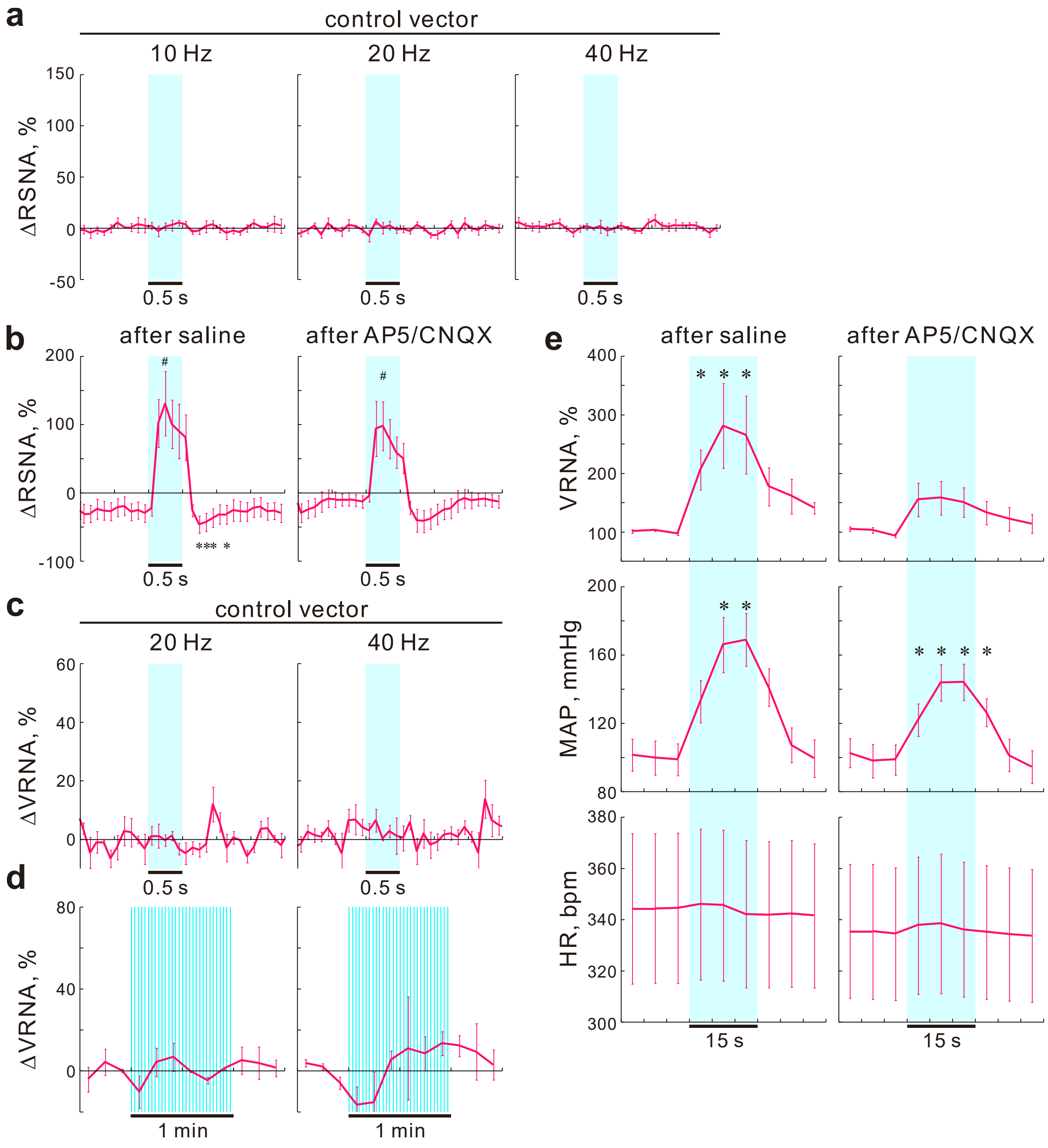

Supplementary Figure 3. MLR→RVLM pathway drives both symapthoexcitation and motor neuroexcitation via glutamatergic transmission. a Time courses (100-ms bins) of ΔRSNA, after superimposing and averaging analysis, in response to intermittent stimulation of MLR→RVLM neurons in EGFP-expressing anesthetized male controls (*n* = 5). **b** Time courses (100-ms bins) of ΔRSNA, after superimposing and averaging analysis, in response to 1-min intermittent stimulation of MLR→RVLM neurons (20 Hz) after bilateral injections into the RVLM of saline or AP5/CNQX in ChR2-GFP-expressing anesthetized male rats (*n* = 6). **c, d** Time courses of ΔVRNA, after superimposing and averaging analysis (c, 100-ms bins) and throughout optogenetic interventions (d, 10-s bins), in response to 1-min intermittent stimulation of the MLR-RVLM neurons in EGFP-expressing decerebrated male controls (*n* = 3). e Time course (5-s bins) changes of VRNA, MAP, and HR during 15-s sustained stimulation of MLR→RVLM neurons (40 Hz) after bilateral injections into the RVLM of saline or AP5/CNQX in ChR2-GFP-expressing decerebrated male rats (*n* = 6). Data were analyzed using one-way RM ANOVA/Friedman

one-way RM ANOVA by rank, followed by Dunnett's *post hoc* test. Statistic information including statistic values and degrees of freedom is presented in Supplementary Table 15. *$P < 0.05$ vs. baseline [averaged over 30 s (b) or 15 s (e)]. #$P < 0.05$ vs. 1-s averaged values immediately before each photostimulation over 30 interventions (b). Baseline values for panels a, b, c/d, and e are reported in Supplementary Tables 3, 4, 6, and 7, respectively. Data shown are means ± SEM. Source data are provided as a Source Data file.

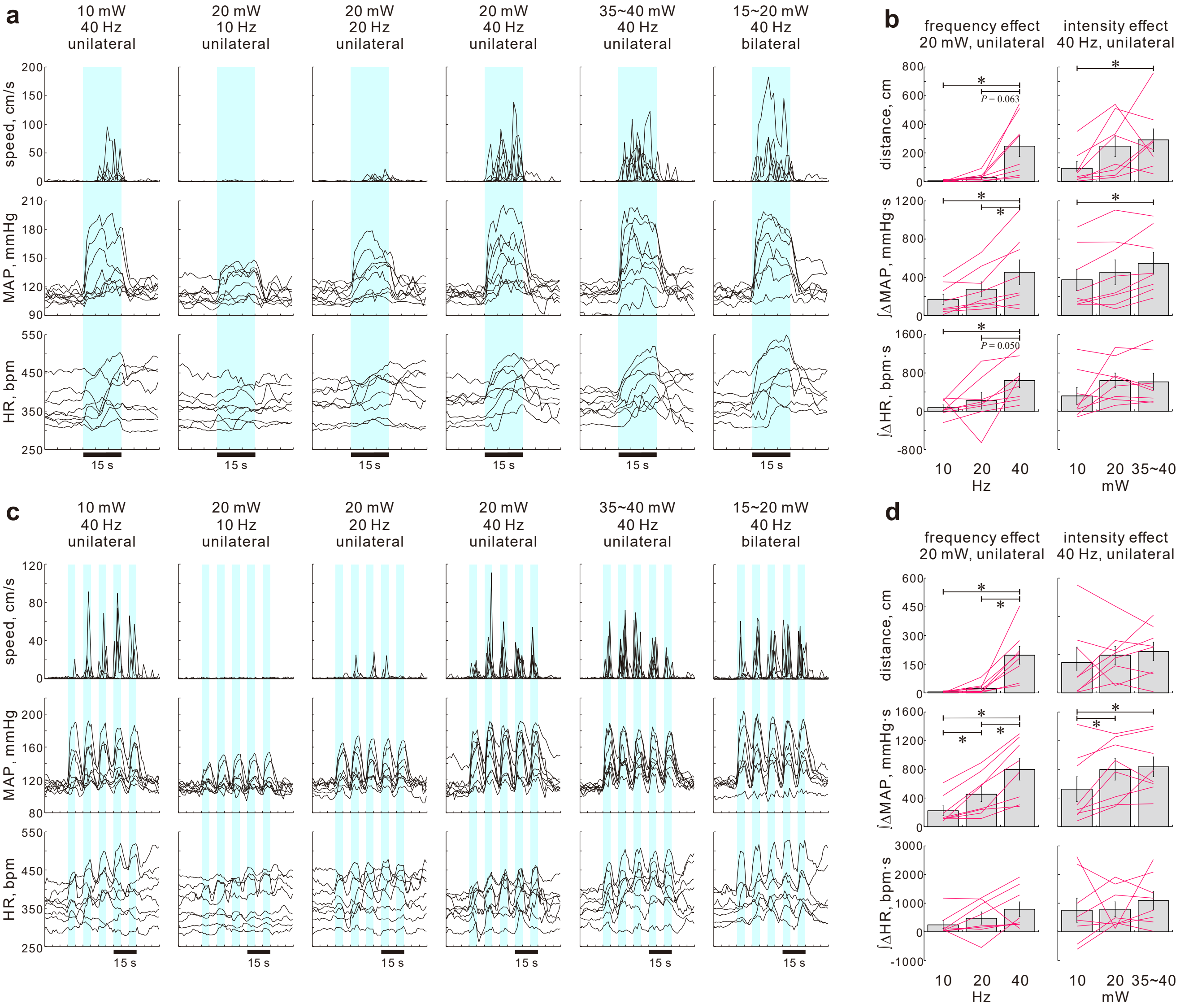

Supplementary Figure 4. MLR→RVLM pathway drives both pressor and locomotor responses. a Individual time courses (1-s bins) of movement speed, AP and HR changes in response to 15-s sustained optogenetic stimulation (n = 8, besides n = 7 for bilateral stimulation, all males). **b** Comparisons of distances ChR2-expressing rats walked/ran and cardiovascular changes during 15-s sustained optogenetic stimulation among optogenetic stimulation frequencies and laser intensities. Locomotor distances and cardiovascular change indexes were obtained from integrated values during 15-s period of laser pulses. **c** Individual time courses (1-s bins) during five-times-repeated intermittent (5-s laser-on/5-s laser-off) optogenetic interventions (n = 8, besides n = 7 for bilateral stimulation). **d** Comparisons of distances walked/ran by ChR2-expressing rats and cardiovascular changes during five-times-repeated optogenetic interventions among optogenetic stimulation frequencies and laser intensities. Locomotor distances and cardiovascular change indexes were obtained from integrated values during the period of laser pulses totally for 25 s. Data were analyzed via one-way RM ANOVA/Friedman one-way RM ANOVA by rank followed by Tukey's *post hoc* test (b, d). Statistic information including statistic values and degrees of freedom is presented in Supplementary Table 15. *P < 0.05 between trials. Baseline values of a/b and c/d are reported in Supplementary Tables 8 and 9, respectively. Data shown are means ± SEM. Source data are provided as a Source Data file.

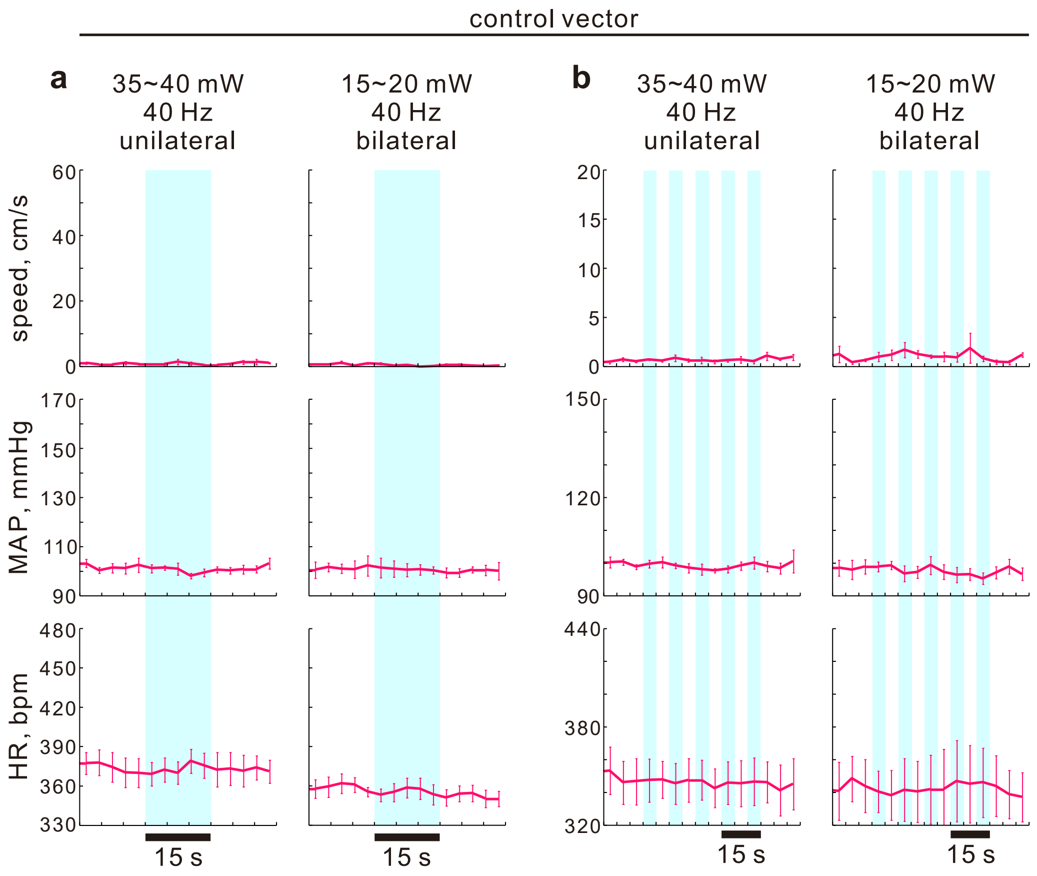

Supplementary Figure 5. Optogenetic interventions of the MLR→RVLM pathway in control rats do not cause locomotion or cardiovascular changes. Time courses of movement speed, AP and HR changes during 15-s sustained (**a**) or five-times-repeated (**b**) optogenetic intervention in EGFP-expressing male control rats (n = 4). Data were analyzed via one-way RM ANOVA/Friedman one-way RM ANOVA by rank. Statistic information including statistic values and degrees of freedom is presented in Supplementary Table 15. Baseline values for a and b are reported in Supplementary Tables 10 and 11, respectively. Data shown are means ± SEM. Source data are provided as a Source Data file.

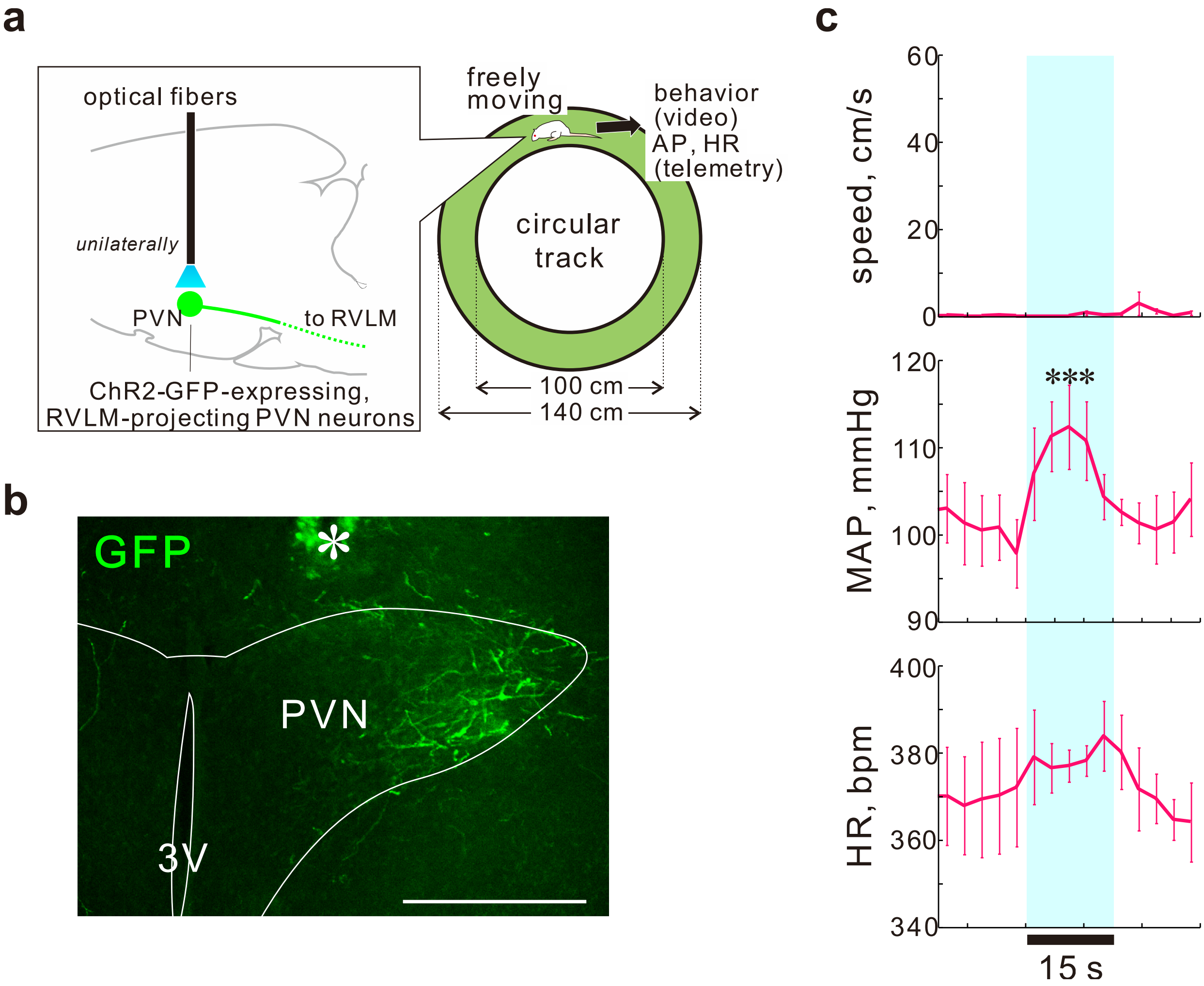

Supplementary Figure 6. PVN→RVLM pathway does not drive locomotion but elicits pressor responses. a Optogenetic stimulation of PVN→RVLM neurons in conscious rats, which were left to move freely in a circular track. **b** GFP-immunoreactive cells in the PVN. Asterisk: implanted optical-fiber tip location. Scale bar: 500 µm. 3V: third ventricle. **c** Time courses (3-s bins) of movement speed, AP and HR changes during 15-s sustained optogenetic intervention (40 Hz; 20 mW) in ChR2-GFP-expressing male rats (n = 5). Data were analyzed using one-way RM ANOVA/Friedman one-way RM ANOVA by rank followed by Dunnett's *post hoc* test . Statistic information including statistic values and degrees of freedom is presented in Supplementary Table 15. *$P < 0.05$ vs. 15-s averaged baseline. Baseline values for c are reported in Supplementary Table 12. Data shown are means ± SEM. Source data are provided as a Source Data file. The brain section image used in the figure (**a**) was adapted from "Paxinos G & Watson C. The rat brain in stereotaxic coordinates. 6th edn. (Amsterdam, Academic Press/Elsevier, 2007)".

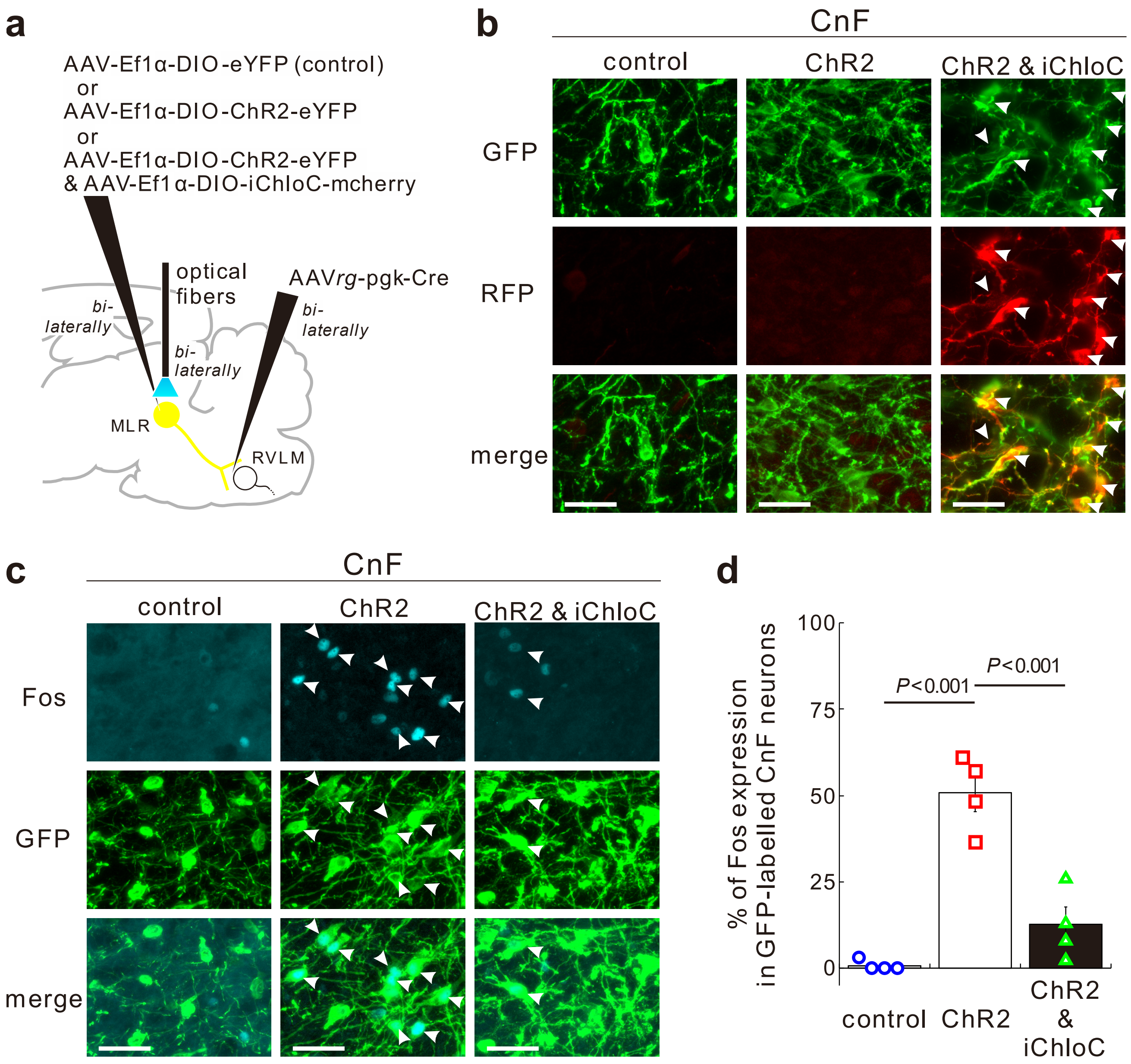

Supplementary Figure 7. ChR2-mediated photoactivation of MLR→RVLM neurons is suppressed by co-activation of iChloC. a Bilateral AAV injections into the MLR and RVLM. **b, c** Immunofluorescence staining of GFP (representing eYFP expression) and RFP (representing mCherry expression) (b) and Fos and GFP (c) in the CnF. Arrowheads indicate RFP (b) or Fos (c) expression in GFP-labeled cells. In rats expressing both ChR2-eYFP and iChloC-mCherry, RFP immunoreactivity was exhibited by 81 ± 8% of GFP-labelled CnF neurons. Scale bars: 50 μm. **d** Comparisons of Fos immunoreactive cells in GFP-labelled, RVLM-projecting CnF neurons among control rats, ChR2-expressing rats, and rats expressing both ChR2 and iChloC [*n* = 4 (3 males and 1 female) for each group]. Data were analyzed by one-way ANOVA followed by Tukey's test. Statistic information including statistic values and degrees of freedom is presented in Supplementary Table 15. Source data are provided as a Source Data file. The brain section image used in the figure (**a**) was adapted from "Paxinos G & Watson C. The rat brain in stereotaxic coordinates. 6th edn. (Amsterdam, Academic Press/Elsevier, 2007)".

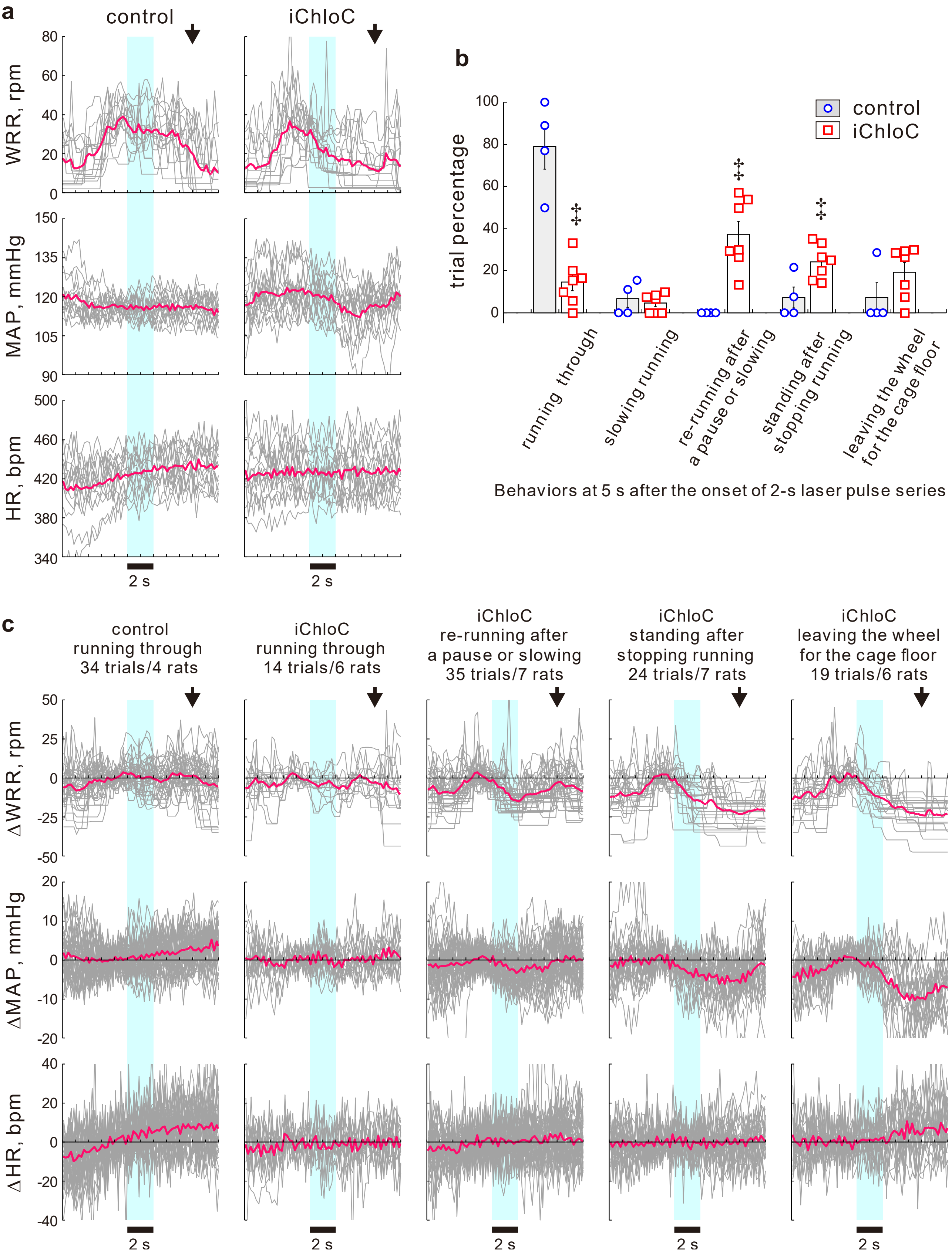
a
control
iChloC
WRR, rpm
MAP, mmHg
HR, bpm
2 s
b
trial percentage
control
iChloC
running through
slowing running
re-running after a pause or slowing
standing after stopping running
leaving the wheel for the cage floor
Behaviors at 5 s after the onset of 2-s laser pulse series
c
control running through 34 trials/4 rats
iChloC running through 14 trials/6 rats
iChloC re-running after a pause or slowing 35 trials/7 rats
iChloC standing after stopping running 24 trials/7 rats
iChloC leaving the wheel for the cage floor 19 trials/6 rats
ΔWRR, rpm
ΔMAP, mmHg
ΔHR, bpm
2 s

Supplementary Figure 8. Locomotor and cardiovascular changes by optogenetic inhibition of the MLR→RVLM pathway during voluntary wheel running. a Individual examples of time course (200-ms bins) changes in each trial (gray) and averaged time courses over the trials (magenta) in eYFP-expressing control and iChloC-mCherry-expressing rats. Data presented were obtained from 9 trials performed over two experimental days in a control rat and 16 trials over three days in an iChloC-mCherry-expressing rat. **b** Comparisons of trial percentages between male controls (n = 4) and iChloC-mCherry-expressing male rats (n = 7) and among behavioral patterns at 5 s after the onset of 2-s laser pulse series (indicated by arrows in a and c). "Slowing running" was defined if running was changed to walking during the 5-s period. Data were analyzed by two-way RM ANOVA followed by Tukey's test. Statistic information including statistic values and degrees of freedom is presented in Supplementary Table 15. ‡$P < 0.05$ vs. controls in each pattern. Data shown are means ± SEM. **c** Superimposed time course changes from pre-illumination level in each behavior 5 s after the onset of 2-s laser pulse series (gray) and averaged time courses over the trials (magenta). The data are presented if the behavior was observed more than 10 trials. Source data are provided as a Source Data file.

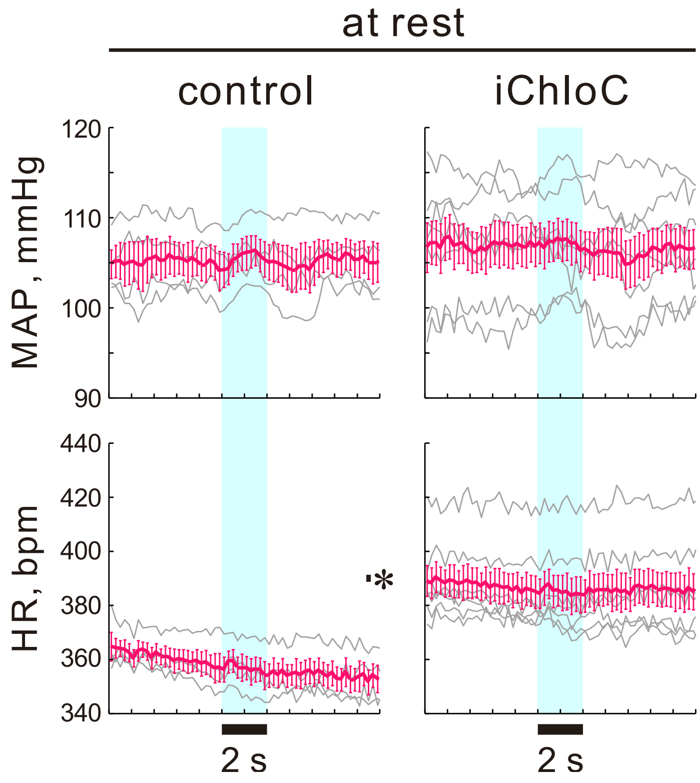

Supplementary Figure 9. Optogenetic inhibition of the MLR→RVLM pathway at rest has no effect on AP or HR. Time course (200-ms bins) changes in response to 2-s optogenetic intervention at rest in eYFP-expressing control rats (*n* = 4) and iChloC-mcherry-expressing male rats (*n* = 7). Gray, individual data after averaging over trials. Data were analyzed by one-way RM ANOVA followed by Holm-Sidak's *post hoc* test/Friedman one-way RM ANOVA by rank followed by Dunnett's *post hoc* test. Statistic information including statistic values and degrees of freedom is presented in Supplementary Table 15. *$P < 0.05$ vs. 2-s averaged pre-illumination level immediately prior to optogenetic intervention. Baseline values (= pre-illumination level) are reported in Supplementary Table 14. Source data are provided as a Source Data file.

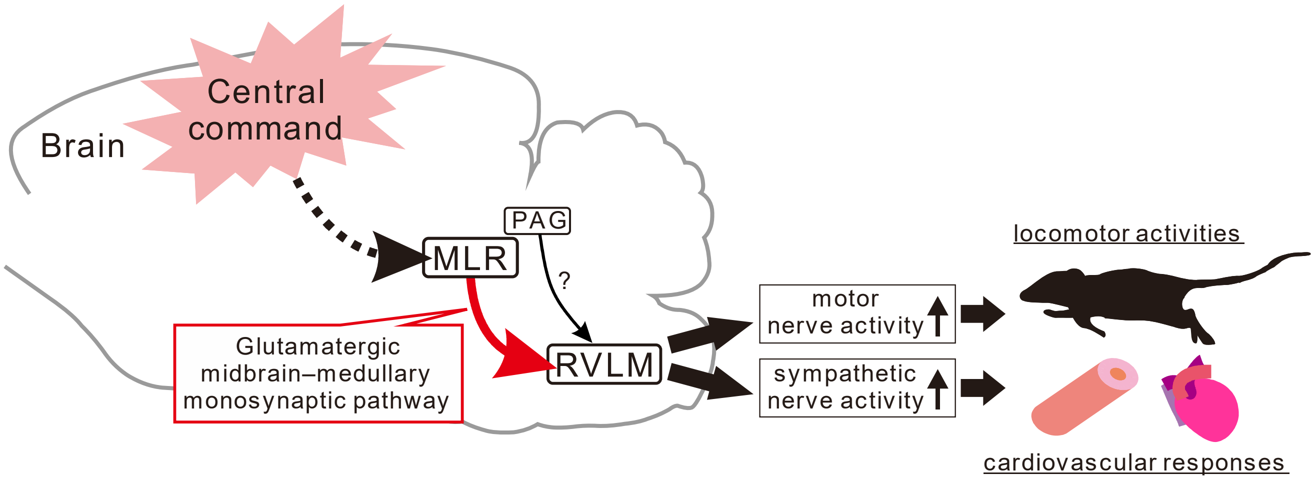

Supplementary Figure 10. Schematic central circuit mechanisms that cause parallel activation of somatomotor and autonomic nervous systems engaged for running exercise. Forebrain site(s) that serve as the origin of central command signals for locomotion or running exercise activate the MLR through unknown pathways. The excited MLR neurons drive both somatic and sympathetic outflow through the RVLM and thereby elicit locomotor activities and cardiovascular responses. The brain section image used in this figure was adapted from ”Paxinos G & Watson C. The rat brain in stereotaxic coordinates. 6th edn. (Amsterdam, Academic Press/Elsevier, 2007)”.

Supplementary Table 1. Baseline values, corresponding to Supplementary Fig. 2c

rat group	control		ChIEF	
trials	20 Hz	40 Hz	20 Hz	40 Hz
# of rats	4 (all males)		7 (all males)	
MAP, mmHg	95 ± 5	96 ± 5	94 ± 4	94 ± 3
HR, bpm	376 ± 22	370 ± 21	323 ± 15	324 ± 13

Supplementary Table 2. Baseline values, corresponding to Fig. 2d

trials	10 Hz	20 Hz	40 Hz
# of rats	7 (5 males, 2 females) *	9 (7 males, 2 females)	
SNR for RSNA	3.17 ± 0.30	3.17 ± 0.23	3.12 ± 0.28
MAP, mmHg	98 ± 4	100 ± 5	101 ± 5
HR, bpm	336 ± 10	338 ± 9	337 ± 9

* Seven of the 9 rats for 20/40 Hz experiments were used for 10 Hz experiments.

Supplementary Table 3. Baseline values, corresponding to Supplementary Fig. 3a

trials	10 Hz	20 Hz	40 Hz
# of rats	5 (all males)		
SNR for RSNA	3.14 ± 0.42	3.09 ± 0.43	2.93 ± 0.44
MAP, mmHg	104 ± 5	99 ± 6	101 ± 6
HR, bpm	396 ± 13	401 ± 10	393 ± 12

Supplementary Table 4. Baseline values, corresponding to Fig. 2e, Supplementary Fig. 3b

trials	saline	AP5/CNQX
# of rats	6 (all males)	
SNR for RSNA	3.91 ± 0.77	4.03 ± 0.60
MAP, mmHg	92 ± 4	96 ± 9
HR, bpm	331 ± 16	342 ± 12

Supplementary Table 5. Baseline values, corresponding to Fig 3c, d

trials	20 Hz	40 Hz
# of rats	6 (all males)	
SNR for RSNA	4.86 ± 0.68	4.92 ± 0.68
SNR for VRNA	5.00 ± 1.38	5.54 ± 1.90
MAP, mmHg	95 ± 9	96 ± 11
HR, bpm	322 ± 25	328 ± 21

Supplementary Table 6. Baseline values, corresponding to Supplementary Fig.3c, d

trials	20 Hz	40 Hz
# of rats	3 (all males)	
SNR for VRNA	1.99 ± 0.50	1.86 ± 0.51
MAP, mmHg	112 ± 15	114 ± 15
HR, bpm	319 ± 32	325 ± 39

Supplementary Table 7. Baseline values, corresponding to Fig 3e, Supplementary Fig. 3e

trials	saline	AP5/CNQX
# of rats	6 (all males)	
SNR for VRNA	3.09 ± 0.64	2.64 ± 0.52
MAP, mmHg	100 ± 10	100 ± 9
HR, bpm	344 ± 29	335 ± 26

Supplementary Table 8. Baseline values, corresponding to Fig 4e, Supplementary Fig. 4a, b

trials	10 mW 40 Hz unilateral	20 mW 10 Hz unilateral	20 mW 20 Hz unilateral	20 mW 40 Hz unilateral	35~40 mW 40 Hz unilateral	15~20 mW 40 Hz bilateral
# of rats	8 (all males)					7*
behavioral speed, cm/s	0.40 ± 0.23	0.15 ± 0.08	0.45 ± 0.14	0.31 ± 0.09	0.29 ± 0.09	0.25 ± 0.10
MAP, mmHg	110 ± 2	111 ± 3	112 ± 3	112 ± 2	112 ± 4	112 ± 2
HR, bpm	360 ± 17	359 ± 17	372 ± 16	365 ± 14	366 ± 11	369 ± 16

* Seven of the 8 rats were used for "bilateral" experiments.

Supplementary Table 9. Baseline values, corresponding to Fig 4g, Supplementary Fig. 4c, d

trials	10 mW 40 Hz unilateral	20 mW 10 Hz unilateral	20 mW 20 Hz unilateral	20 mW 40 Hz unilateral	35~40 mW 40 Hz unilateral	15~20 mW 40 Hz bilateral
# of rats	8 (all males)					7*
behavioral speed, cm/s	0.43 ± 0.11	0.17 ± 0.04	0.23 ± 0.05	0.38 ± 0.16	0.20 ± 0.08	0.42 ± 0.17
MAP, mmHg	112 ± 2	110 ± 1	112 ± 2	112 ± 5	110 ± 1	114 ± 3
HR, bpm	355 ± 14	356 ± 18	366 ± 18	346 ± 11	356 ± 12	351 ± 14

* Seven of the 8 rats were used for "bilateral" experiments.

Supplementary Table 10. Baseline values, corresponding to Supplementary Fig. 5a

trials	35~40 mW 40 Hz unilateral	15~20 mW 40 Hz bilateral
# of rats	4 (all males)	
behavioral speed, cm/s	0.53 ± 0.28	0.79 ± 0.25
MAP, mmHg	102 ± 2	101 ± 3
HR, bpm	374 ± 9	359 ± 6

Supplementary Table 11. Baseline values, corresponding to Supplementary Fig. 5b

trials	35~40 mW<br>40 Hz<br>unilateral	15~20 mW<br>40 Hz<br>bilateral
# of rats	4 (all males)	
behavioral speed, cm/s	0.54 ± 0.14	0.75 ± 0.34
MAP, mmHg	100 ± 1	98 ± 2
HR, bpm	349 ± 14	344 ± 16

Supplementary Table 12. Baseline values, corresponding to Supplementary Fig. 6c

# of rats	5 (all males)
behavioral speed, cm/s	0.14 ± 0.08
MAP, mmHg	101 ± 4
HR, bpm	370 ± 12

Supplementary Table 13. Baseline values, corresponding to Fig 5e-h

rat group	control	iChloC
# of rats	4 (all males)	7 (all males)
# of trials	11.3 ± 1.3	13.7 ± 0.9
WRR, rpm	32.5 ± 2.4	27.9 ± 1.3
MAP, mmHg	113 ± 2	119 ± 2
HR, bpm	4316	436 ± 8

Supplementary Table 14. Baseline values, corresponding to Fig 5i, Supplementary Fig. 9

rat group	control	iChloC
# of rats	4 (all males)	7 (all males)
# of trials	9.8 ± 0.8	9.9 ± 1.5
MAP, mmHg	105 ± 2	107 ± 2
HR, bpm	359 ± 4	386 ± 6

Supplementary Table 15. Detailed statistical information.

Figure#	Data	# of animals	Normality/equal variance test	Primary statistic	Post-hoc test, two-sided	Comparison	p value	F/t/χ2 statistic value
1k	Mean percentrage of Fos expression per GFP-positive population	14, 7/group	Normality Test (Shapiro-Wilk): Passed (P = 0.054) Equal Variance Test (Brown-Forsythe): Passed (P = 0.359)	Welch's t-test, two-sided		non-Ex control vs. Ex	0.0392	t = -2.358 with 10.390 degrees of freedom
	Mean number of Fos expression in GFP-positive population	14, 7/group	Normality Test (Shapiro-Wilk): Passed (P = 0.076) Equal Variance Test (Brown-Forsythe): Passed (P = 0.097)	Welch's t-test, two-sided		non-Ex control vs. Ex	0.0406	t = -2.535 with 6.670 degrees of freedom.
2d	Time course of ΔRSNA (10 Hz), superimposing and averaging analysis.	7	Normality Test (Shapiro-Wilk): Failed ($P < 0.050$)	Friedman Repeated Measures Analysis of Variance on Ranks		Main effect of time	<0.001	$\chi2$= 108.487 with 31 degrees of freedom.
					Dunnett's Method	vs. 30 s-averaged baseline	See Fig	

					Dunnett's Method	vs. 1 s-averaged values immediately prior to each photostim.	See Fig	
	Time course of ΔRSNA (20 Hz), superimposing and averaging analysis.	9	Normality Test (Shapiro-Wilk): Failed (P < 0.050)	Friedman Repeated Measures Analysis of Variance on Ranks		Main effect of time	<0.001	χ2= 134.980 with 31 degrees of freedom.
					Dunnett's Method	vs. 30 s-averaged baseline	See Fig	
					Dunnett's Method	vs. 1 s-averaged values immediately prior to each photostim.	See Fig	
	Time course of ΔRSNA (40 Hz), superimposing and averaging analysis.	9	Normality Test (Shapiro-Wilk): Failed (P < 0.050)	Friedman Repeated Measures Analysis of Variance on Ranks		Main effect of time	<0.001	χ2= 85.215 with 31 degrees of freedom.
					Dunnett's Method	vs. 30 s-averaged baseline	See Fig	

					Dunnett's Method	vs. 1 s-averaged values immediately prior to each photostim.	See Fig	
2e	AUC of ΔRSNA	6	Normality Test (Shapiro-Wilk): Passed (P = 0.294)	Paired t-test, two-sided		saline vs. AP5/CNQX	0.0393	t = 2.772 with 5 degrees of freedom.
3c	Time course of ΔRSNA (20 Hz), superimposing and averaging analysis.	6	Normality Test (Shapiro-Wilk): Failed (P < 0.050)	Friedman Repeated Measures Analysis of Variance on Ranks		Main effect of time	<0.001	$\chi2$= 109.303 with 31 degrees of freedom.
					Dunnett's Method	vs. 30 s-averaged baseline	See Fig	
					Dunnett's Method	vs. 1 s-averaged values immediately prior to each photostim.	See Fig	
	Time course of ΔVRNA (20 Hz), superimposing and averaging analysis.	6	Normality Test (Shapiro-Wilk): Passed (P = 0.056) Equal Variance Test (Brown-Forsythe):	One Way Repeated Measures Analysis of Variance		Main effect of time	<0.001	F = 2.286 with 31 degrees of freedom.

			Passed (P = 0.270)					
					Dunnett's Method	vs. 30 s-averaged baseline	See Fig	
					Dunnett's Method	vs. 1 s-averaged values immediately prior to each photostim.	See Fig	
	Time course of ΔRSNA (40 Hz), superimposing and averaging analysis.	6	Normality Test (Shapiro-Wilk): Failed (P < 0.050)	Friedman Repeated Measures Analysis of Variance on Ranks		Main effect of time	<0.001	$\chi 2$= 146.106 with 31 degrees of freedom.
					Dunnett's Method	vs. 30 s-averaged baseline	See Fig	
					Dunnett's Method	vs. 1 s-averaged values immediately prior to each photostim.	See Fig	

	Time course of ΔVRNA (40 Hz), superimposing and averaging analysis.	6	Normality Test (Shapiro-Wilk): Failed ($P < 0.050$)	Friedman Repeated Measures Analysis of Variance on Ranks		Main effect of time	0.123	χ^2= 40.292 with 31 degrees of freedom.
3d	Time course of ΔRSNA (20 Hz), 10 s-averaged time course	6	Normality Test (Shapiro-Wilk): Failed ($P < 0.050$)	Friedman Repeated Measures Analysis of Variance on Ranks		Main effect of time	0.158	χ^2= 16.791 with 12 degrees of freedom.
	Time course of ΔVRNA (20 Hz), 10 s-averaged time course	6	Normality Test (Shapiro-Wilk): Failed ($P < 0.050$)	Friedman Repeated Measures Analysis of Variance on Ranks		Main effect of time	0.004	χ^2= 29.209 with 12 degrees of freedom.
					Dunnett's Method	vs. 30 s-averaged baseline	See Fig	
	Time course of ΔRSNA (40 Hz), 10 s-averaged time course	6	Normality Test (Shapiro-Wilk): Passed ($P = 0.349$) Equal Variance Test (Brown-	One Way Repeated Measures Analysis of Variance		Main effect of time	0.452	F = 1.010 with 12 degrees of freedom.

			Forsythe): Passed (P = 0.976)					
	Time course of ΔVRNA (40 Hz), 10 s-averaged time course	6	Normality Test (Shapiro-Wilk): Failed (P < 0.050)	Friedman Repeated Measures Analysis of Variance on Ranks		Main effect of time	<0.001	$\chi2$= 50.000 with 12 degrees of freedom.
					Dunnett's Method	vs. 30 s-averaged baseline	See Fig	
3e	Integrated ΔVRNA	6	Normality Test (Shapiro-Wilk): Passed (P = 0.186)	Paired t-test, two-sided		saline vs. AP5/CNQX	0.0251	t = 3.162 with 5 degrees of freedom.
	Integrated ΔMAP	6	Normality Test (Shapiro-Wilk): Passed (P = 0.189)	Paired t-test, two-sided		saline vs. AP5/CNQX	0.0083	t = 4.223 with 5 degrees of freedom.
4e	Speed, 10 mW, 40 Hz, unilateral	8	Normality Test (Shapiro-Wilk): Failed (P < 0.050)	Friedman Repeated Measures Analysis of		Main effect of time	0.003	$\chi2$= 34.170 with 15 degrees of freedom.

				Variance on Ranks				
					Dunnett's Method	vs. 15 s-averaged baseline	See Fig	
	MAP, 10 mW, 40 Hz, unilateral	8	Normality Test (Shapiro-Wilk): Failed (P < 0.050)	Friedman Repeated Measures Analysis of Variance on Ranks		Main effect of time	<0.001	χ2= 98.901 with 15 degrees of freedom.
					Dunnett's Method	vs. 15 s-averaged baseline	See Fig	
	HR, 10 mW, 40 Hz, unilateral	8	Normality Test (Shapiro-Wilk): Failed (P < 0.050)	Friedman Repeated Measures Analysis of Variance on Ranks		Main effect of time	0.037	χ2= 26.140 with 15 degrees of freedom.
					Dunnett's Method	vs. 15 s-averaged baseline	See Fig	
	Speed, 20 mW, 10 Hz, unilateral	8	Normality Test (Shapiro-Wilk): Failed (P < 0.050)	Friedman Repeated Measures Analysis of Variance s of		Main effect of time	0.187	χ2= 19.615 with 15 degrees of freedom.

				Variance on Ranks				
	MAP, 20 mW, 10 Hz, unilateral	8	Normality Test (Shapiro-Wilk): Passed (P = 0.074) Equal Variance Test (Brown-Forsythe): Failed (P < 0.050)	Friedman Repeated Measures Analysis of Variance on Ranks		Main effect of time	<0.001	χ2= 64.235 with 15 degrees of freedom.
					Dunnett's Method	vs. 15 s-averaged baseline	See Fig	
	HR, 20 mW, 10 Hz, unilateral	8	Normality Test (Shapiro-Wilk): Failed (P < 0.050)	Friedman Repeated Measures Analysis of Variance on Ranks		Main effect of time	0.433	χ2= 15.254 with 15 degrees of freedom.
	Speed, 20 mW, 20 Hz, unilateral	8	Normality Test (Shapiro-Wilk): Failed (P < 0.050)	Friedman Repeated Measures Analysis of Variance on Ranks		Main effect of time	0.02	χ2= 28.177 with 15 degrees of freedom.
					Dunnett's	vs. 15 s-averaged baseline	See Fig	

					Method			
	MAP, 20 mW, 20 Hz, unilateral	8	Normality Test (Shapiro-Wilk): Failed (P < 0.050)	Friedman Repeated Measures Analysis of Variance on Ranks		Main effect of time	<0.001	χ2= 90.210 with 15 degrees of freedom.
					Dunnett's Method	vs. 15 s-averaged baseline	See Fig	
	HR, 20 mW, 20 Hz, unilateral	8	Normality Test (Shapiro-Wilk): Failed (P < 0.050)	Friedman Repeated Measures Analysis of Variance on Ranks		Main effect of time	0.003	χ2= 34.169 with 15 degrees of freedom.
					Dunnett's Method	vs. 15 s-averaged baseline	See Fig	
	Speed, 20 mW, 40 Hz, unilateral	8	Normality Test (Shapiro-Wilk): Failed (P < 0.050)	Friedman Repeated Measures Analysis of Variance on Ranks		Main effect of time	<0.001	χ2= 72.252 with 15 degrees of freedom.
					Dunnett's Method	vs. 15 s-averaged baseline	See Fig	

	MAP, 20 mW, 40 Hz, unilateral	8	Normality Test (Shapiro-Wilk): Failed (P < 0.050)	Friedman Repeated Measures Analysis of Variance on Ranks		Main effect of time	<0.001	χ2= 82.721 with 15 degrees of freedom.
					Dunnett's Method	vs. 15 s-averaged baseline	See Fig	
	HR, 20 mW, 40 Hz, unilateral	8	Normality Test (Shapiro-Wilk): Passed (P = 0.652) Equal Variance Test (Brown-Forsythe): Passed (P = 0.993)	One Way Repeated Measures Analysis of Variance		Main effect of time	<0.001	F = 11.120 with 15 degrees of freedom.
					Dunnett's Method	vs. 15 s-averaged baseline	See Fig	
	Speed, 35~40 mW, 40 Hz, unilateral	8	Normality Test (Shapiro-Wilk): Failed (P < 0.050)	Friedman Repeated Measures Analysis of Variance on Ranks		Main effect of time	<0.001	χ2= 58.291 with 15 degrees of freedom.
					Dunnett's Method	vs. 15 s-averaged baseline	See Fig	

	MAP, 35~40 mW, 40 Hz, unilateral	8	Normality Test (Shapiro-Wilk): Passed (P = 0.169) Equal Variance Test (Brown-Forsythe): Passed (P = 0.056)	One Way Repeated Measures Analysis of Variance		Main effect of time	<0.001	F = 14.53120 with 15 degrees of freedom.
					Dunnett's Method	vs. 15 s-averaged baseline	See Fig	
	HR, 35~40 mW, 40 Hz, unilateral	8	Normality Test (Shapiro-Wilk): Failed (P < 0.050)	Friedman Repeated Measures Analysis of Variance on Ranks		Main effect of time	<0.001	$\chi2$= 62.482 with 15 degrees of freedom.
					Dunnett's Method	vs. 15 s-averaged baseline	See Fig	
	Speed, 15~20 mW, 40 Hz, bilateral	7	Normality Test (Shapiro-Wilk): Failed (P < 0.050)	Friedman Repeated Measures Analysis of Variance on Ranks		Main effect of time	<0.001	$\chi2$= 66.530 with 15 degrees of freedom.
					Dunnett's Method	vs. 15 s-averaged baseline	See Fig	

	MAP, 15~20 mW, 40 Hz, bilateral	7	Normality Test (Shapiro-Wilk): Passed (P = 0.523) Equal Variance Test (Brown-Forsythe): Passed (P = 0.134)	One Way Repeated Measures Analysis of Variance		Main effect of time	<0.001	F = 14.633 with 15 degrees of freedom.
					Dunnett's Method	vs. 15 s-averaged baseline	See Fig	
	HR, 15~20 mW, 40 Hz, bilateral	7	Normality Test (Shapiro-Wilk): Passed (P = 0.523) Equal Variance Test (Brown-Forsythe): Passed (P = 0.134)	One Way Repeated Measures Analysis of Variance		Main effect of time	<0.001	F = 5.748 with 15 degrees of freedom.
					Dunnett's Method	vs. 15 s-averaged baseline	See Fig	
4g	Speed, 10 mW, 40 Hz, unilateral	8	Normality Test (Shapiro-Wilk): Failed (P < 0.050)	Friedman Repeated Measures Analysis of Variance on Ranks		Main effect of time	0.047	$\chi2$= 25.260 with 15 degrees of freedom.

					Dunnett's Method	vs. 15 s-averaged baseline	See Fig	
	MAP, 10 mW, 40 Hz, unilateral	8	Normality Test (Shapiro-Wilk): Failed (P < 0.050)	Friedman Repeated Measures Analysis of Variance on Ranks		Main effect of time	<0.001	χ2= 88.191 with 15 degrees of freedom.
					Dunnett's Method	vs. 15 s-averaged baseline	See Fig	
	HR, 10 mW, 40 Hz, unilateral	8	Normality Test (Shapiro-Wilk): Passed (P = 0.599) Equal Variance Test (Brown-Forsythe): Passed (P = 0.972)	One Way Repeated Measures Analysis of Variance		Main effect of time	0.026	F =1.949 with 15 degrees of freedom.
					Dunnett's Method	vs. 15 s-averaged baseline	See Fig	
	Speed, 20 mW, 10 Hz, unilateral	8	Normality Test (Shapiro-Wilk): Failed (P < 0.050)	Friedman Repeated Measures Analysis of Variance on Ranks		Main effect of time	0.134	χ2= 21.084 with 15 degrees of freedom.

	MAP, 20 mW, 10 Hz, unilateral	8	Normality Test (Shapiro-Wilk): Failed (P < 0.050)	Friedman Repeated Measures Analysis of Variance on Ranks		Main effect of time	<0.001	χ2= 65.537 with 15 degrees of freedom.
					Dunnett's Method	vs. 15 s-averaged baseline	See Fig	
	HR, 20 mW, 10 Hz, unilateral	8	Normality Test (Shapiro-Wilk): Passed (P = 0.276) Equal Variance Test (Brown-Forsythe): Passed (P = 0.903)	One Way Repeated Measures Analysis of Variance		Main effect of time	<0.001	F =3.459 with 15 degrees of freedom.
					Dunnett's Method	vs. 15 s-averaged baseline	See Fig	
	Speed, 20 mW, 20 Hz, unilateral	8	Normality Test (Shapiro-Wilk): Failed (P < 0.050)	Friedman Repeated Measures Analysis of Variance on Ranks		Main effect of time	0.627	χ2= 12.677 with 15 degrees of freedom.

	MAP, 20 mW, 20 Hz, unilateral	8	Normality Test (Shapiro-Wilk): Failed (P < 0.050)	Friedman Repeated Measures Analysis of Variance on Ranks		Main effect of time	<0.001	$\chi2$= 86.129 with 15 degrees of freedom.
					Dunnett's Method	vs. 15 s-averaged baseline	See Fig	
	HR, 20 mW, 20 Hz, unilateral	8	Normality Test (Shapiro-Wilk): Passed (P = 0.144) Equal Variance Test (Brown-Forsythe): Passed (P = 0.889)	One Way Repeated Measures Analysis of Variance		Main effect of time	<0.001	F =2.730 with 15 degrees of freedom.
					Dunnett's Method	vs. 15 s-averaged baseline	See Fig	
	Speed, 20 mW, 40 Hz, unilateral	8	Normality Test (Shapiro-Wilk): Failed (P < 0.050)	Friedman Repeated Measures Analysis of Variance on Ranks		Main effect of time	<0.001	$\chi2$= 55.134 with 15 degrees of freedom.
					Dunnett's Method	vs. 15 s-averaged baseline	See Fig	

	MAP, 20 mW, 40 Hz, unilateral	8	Normality Test (Shapiro-Wilk): Passed (P = 0.127) Equal Variance Test (Brown-Forsythe): Passed (P = 0.759)	One Way Repeated Measures Analysis of Variance		Main effect of time	<0.001	F = 17.426 with 15 degrees of freedom.
					Dunnett's Method	vs. 15 s-averaged baseline	See Fig	
	HR, 20 mW, 40 Hz, unilateral	8	Normality Test (Shapiro-Wilk): Passed (P = 0.223) Equal Variance Test (Brown-Forsythe): Passed (P = 0.938)	One Way Repeated Measures Analysis of Variance		Main effect of time	<0.001	F = 2.993 with 15 degrees of freedom.
					Dunnett's Method	vs. 15 s-averaged baseline	See Fig	
	Speed, 35~40 mW, 40 Hz, unilateral	8	Normality Test (Shapiro-Wilk): Failed (P < 0.050)	Friedman Repeated Measures Analysis of Variance on Ranks		Main effect of time	<0.001	$\chi2$= 65.497 with 15 degrees of freedom.

					Dunnett's Method	vs. 15 s-averaged baseline	See Fig	
	MAP, 35~40 mW, 40 Hz, unilateral	8	Normality Test (Shapiro-Wilk): Passed (P = 0.456) Equal Variance Test (Brown-Forsythe): Passed (P = 0.450)	One Way Repeated Measures Analysis of Variance		Main effect of time	<0.001	F = 25.128 with 15 degrees of freedom.
					Dunnett's Method	vs. 15 s-averaged baseline	See Fig	
	HR, 35~40 mW, 40 Hz, unilateral	8	Normality Test (Shapiro-Wilk): Failed (P < 0.050)	Friedman Repeated Measures Analysis of Variance on Ranks		Main effect of time	<0.001	$\chi2$= 49.743 with 15 degrees of freedom.
					Dunnett's Method	vs. 15 s-averaged baseline	See Fig	
	Speed, 15~20 mW, 40 Hz, bilateral	7	Normality Test (Shapiro-Wilk): Failed (P < 0.050)	Friedman Repeated Measures Analysis of Variance on Ranks		Main effect of time	<0.001	$\chi2$= 60.908 with 15 degrees of freedom.

					Dunnett's Method	vs. 15 s-averaged baseline	See Fig	
	MAP, 15~20 mW, 40 Hz, bilateral	7	Normality Test (Shapiro-Wilk): Passed (P = 0.156) Equal Variance Test (Brown-Forsythe): Passed (P = 0.647)	One Way Repeated Measures Analysis of Variance		Main effect of time	<0.001	F =11.972 with 15 degrees of freedom.
					Dunnett's Method	vs. 15 s-averaged baseline	See Fig	
	HR, 15~20 mW, 40 Hz, bilateral	7	Normality Test (Shapiro-Wilk): Passed (P = 0.293) Equal Variance Test (Brown-Forsythe): Passed (P = 0.455)	One Way Repeated Measures Analysis of Variance		Main effect of time	<0.001	F = 4.989 with 15 degrees of freedom.
					Dunnett's Method	vs. 15 s-averaged baseline	See Fig	
5e	WRR, control	4	Normality Test (Shapiro-Wilk): Failed (P < 0.050)	Friedman Repeated Measures Analysis of Varianc		Main effect of time	0.077	χ2= 53.345 with 40 degrees of freedom.

				e on Ranks				
	MAP, control	4	Normality Test (Shapiro-Wilk): Passed (P = 0.436) Equal Variance Test (Brown-Forsythe): Passed (P = 0.618)	One Way Repeated Measures Analysis of Variance		Main effect of time	0.016	F = 1.687 with 40 degrees of freedom.
					Dunnett's Method	vs. 2 s-averaged baseline	See Fig	
	HR, control	4	Normality Test (Shapiro-Wilk): Passed (P = 0.840) Equal Variance Test (Brown-Forsythe): Passed (P = 0.194)	One Way Repeated Measures Analysis of Variance		Main effect of time	<0.001	F = 3.627 with 40 degrees of freedom.
					Holm-Sidak’s Method	vs. 2 s-averaged baseline	See Fig	

	WRR, iChloC	7	Normality Test (Shapiro-Wilk): Passed (P = 0.661) Equal Variance Test (Brown-Forsythe): Failed (P < 0.050)	Friedman Repeated Measures Analysis of Variance on Ranks		Main effect of time	<0.001	χ2= 219.524 with 40 degrees of freedom.
					Dunnett's Method	vs. 2 s-averaged baseline	See Fig	
	MAP, iChloC	7	Normality Test (Shapiro-Wilk): Passed (P = 0.325) Equal Variance Test (Brown-Forsythe): Passed (P = 0.990)	One Way Repeated Measures Analysis of Variance		Main effect of time	<0.001	F = 8.411 with 40 degrees of freedom.
					Dunnett's Method	vs. 2 s-averaged baseline	See Fig	
	HR, iChloC	7	Normality Test (Shapiro-Wilk): Passed (P = 0.352) Equal Variance Test (Brown-Forsythe): Passed (P = 0.975)	One Way Repeated Measures Analysis of Variance		Main effect of time	0.295	F = 1.122 with 40 degrees of freedom.

5f	∫ΔWRR	45, control 96, iChloC	Normality Test (Shapiro-Wilk): Passed (P = 0.181) Equal Variance Test (Brown-Forsythe): Passed (P = 0.887)	Welch’s t-test, two-sided		control vs. iChloC	<0.001	t = 6.708 with 90.636 degrees of freedom.
	∫ΔMAP	45, control 96, iChloC	Normality Test (Shapiro-Wilk): Passed (P = 0.091) Equal Variance Test (Brown-Forsythe): Passed (P = 0.353)	Welch’s t-test, two-sided		control vs. iChloC	<0.001	t = 5.188 with 95.389 degrees of freedom.
5h	trial percentages	4, control; 7, iChloC	Normality Test (Shapiro-Wilk): Passed (P = 0.475) Equal Variance Test (Brown-Forsythe): Passed (P = 0.943)	Two Way Repeated Measures ANOVA (One Factor Repetition)		control vs. iChloC, among result patterns of ∫ΔWRR and ∫ΔMAP	result patterns, <0.001; rat group X result patterns, <0.001	F = 29.976 (result patterns of ∫ΔWRR and ∫ΔMAP), F = 33.754 (rat group X result patterns)
					Tukey's Method	all pairs	See Fig	

5i	∫ΔMAP	39, control 69, iChloC	Normality Test (Shapiro-Wilk): Failed ($P < 0.050$)	Mann-Whitney Rank Sum Test		control vs. iChloC	0.341	
Ext. 1d	Mean percentrage of Fos expression per ChAT-positive population	14, 7/group	Normality Test (Shapiro-Wilk): Passed ($P = 0.302$) Equal Variance Test (Brown-Forsythe): Passed ($P = 0.186$)	Welch's t-test		non-Ex control vs. Ex	0.024	$t = -2.780$ with 8.124 degrees of freedom.
	Mean number of Fos expression in ChAT-positive population	14, 7/group	Normality Test (Shapiro-Wilk): Passed ($P = 0.256$) Equal Variance Test (Brown-Forsythe): Failed ($P < 0.050$)	Welch's t-test		non-Ex control vs. Ex	0.0119	$t = -3.447$ with 6.562 degrees of freedom.
Suppl. 2c	MAP, control, 20 Hz	4	Normality Test (Shapiro-Wilk) Passed ($P = 0.250$) Equal Variance Test: Failed ($P < 0.050$)	Friedman Repeated Measures Analysis of Variance on Ranks		Main effect of time	0.562	$\chi 2= 14.490$ with 16 degrees of freedom.

	HR, control, 20 Hz	4	Normality Test (Shapiro-Wilk) Passed (P = 0.393) Equal Variance Test: Passed (P = 0.552)	One Way Repeated Measures Analysis of Variance		Main effect of time	0.534	F = 0.934 with 16 degrees of freedom.
	MAP, control, 40 Hz	4	Normality Test (Shapiro-Wilk) Passed (P = 0.534) Equal Variance Test: Passed (P = 0.969)	One Way Repeated Measures Analysis of Variance		Main effect of time	0.565	F = 0.908 with 16 degrees of freedom.
	HR, control, 40 Hz	4	Normality Test (Shapiro-Wilk) Passed (P = 0.113) Equal Variance Test: Passed (P = 0.279)	One Way Repeated Measures Analysis of Variance		Main effect of time	0.003	F = 2.778 with 16 degrees of freedom.
					Dunnett's Method	vs. 2 s-averaged baseline	See Fig	
	MAP. ChIEF, 20 Hz	7	Normality Test (Shapiro-Wilk) Passed (P = 0.183) Equal Variance Test:	One Way Repeated Measures Analysis of Variance		Main effect of time	<0.001	F = 8.752 with 16 degrees of freedom.

			Passed (P = 0.980)					
					Dunnett's Method	vs. 60 s-averaged baseline	See Fig	
	HR, ChIEF, 20 Hz	7	Normality Test (Shapiro-Wilk) Failed (P < 0.050)	Friedman Repeated Measures Analysis of Variance on Ranks		Main effect of time	0.001	χ2= 38.118 with 16 degrees of freedom.
					Dunnett's Method	vs. 60 s-averaged baseline	See Fig	
	MAP, ChIEF, 40 Hz	7	Normality Test (Shapiro-Wilk) Passed (P = 0.102) Equal Variance Test: Passed (P = 0.396)	One Way Repeated Measures Analysis of Variance		Main effect of time	<0.001	F = 6.970 with 16 degrees of freedom.
					Dunnett's Method	vs. 60 s-averaged baseline	See Fig	

	HR, ChIEF, 40 Hz	7	Normality Test (Shapiro-Wilk) Failed (P < 0.050)	Friedman Repeated Measures Analysis of Variance on Ranks		Main effect of time	<0.001	$\chi2$= 90.902 with 16 degrees of freedom.
					Dunnett's Method	vs. 60 s-averaged baseline	See Fig	
Suppl. 3a	Time course of ΔRSNA (10 Hz), superimposing and averaging analysis.	5	Normality Test (Shapiro-Wilk): Failed (P < 0.050)	Friedman Repeated Measures Analysis of Variance on Ranks		Main effect of time	0.925	$\chi2$ = 20.495 with 31 degrees of freedom.
	Time course of ΔRSNA (20 Hz), superimposing and averaging analysis.	5	Normality Test (Shapiro-Wilk): Passed (P = 0.064) Equal Variance Test (Brown-Forsythe): Passed (P = 0.845)	One Way Repeated Measures Analysis of Variance		Main effect of time	0.484	F = 0.995 with 31 degrees of freedom.

	Time course of ΔRSNA (40 Hz), superimposing and averaging analysis.	5	Normality Test (Shapiro-Wilk): Passed (P = 0.270) Equal Variance Test (Brown-Forsythe): Passed (P = 0.709)	One Way Repeated Measures Analysis of Variance		Main effect of time	0.927	F = 0.636 with 31 degrees of freedom.
Suppl. 3b	Time course of ΔRSNA after saline	6	Normality Test (Shapiro-Wilk): Failed (P < 0.050)	Friedman Repeated Measures Analysis of Variance on Ranks		Main effect of time	<0.001	χ2 = 117.549 with 31 degrees of freedom.
					Dunnett's Method	vs. 30 s-averaged baseline	See Fig	
					Dunnett's Method	vs. 1 s-averaged values immediately prior to each photostim.	See Fig	
	Time course of ΔRSNA after AP5/CNQX	6	Normality Test (Shapiro-Wilk): Failed (P < 0.050)	Friedman Repeated Measures Analysis of Variance on Ranks		Main effect of time	<0.001	χ2 = 104.568 with 31 degrees of freedom.

					Dunnett's Method	vs. 30 s-averaged baseline	See Fig	
					Dunnett's Method	vs. 1 s-averaged values immediately prior to each photostim.	See Fig	
Suppl. 3c	Time course of ΔVRNA (20 Hz), superimposing and averaging analysis.	3	Normality Test (Shapiro-Wilk): Passed (P = 0.967) Equal Variance Test (Brown-Forsythe): Passed (P = 0.491)	One Way Repeated Measures Analysis of Variance		Main effect of time	0.057	F = 1.604 with 31 degrees of freedom.
	Time course of ΔVRNA (40 Hz), superimposing and averaging analysis.	3	Normality Test (Shapiro-Wilk): Passed (P = 0.180) Equal Variance Test (Brown-Forsythe): Passed (P = 0.397)	One Way Repeated Measures Analysis of Variance		Main effect of time	0.218	F = 1.259 with 31 degrees of freedom.
Suppl. 3d	Time course of ΔVRNA (20 Hz), 10 s-averaged time course	3	Normality Test (Shapiro-Wilk): Passed (P = 0.486) Equal Variance Test (Brown-	One Way Repeated Measures Analysis of Variance		Main effect of time	0.683	F = 0.760 with 12 degrees of freedom.

			Forsythe): Passed (P = 0.827)					
		3	Normality Test (Shapiro-Wilk): Passed (P = 0.061) Equal Variance Test (Brown-Forsythe): Passed (P = 0.414)	One Way Repeated Measures Analysis of Variance		Main effect of time	0.545	F = 0.917 with 12 degrees of freedom.
Suppl. 3e	VRNA, after saline	6	Normality Test (Shapiro-Wilk): Passed (P = 0.403) Equal Variance Test (Brown-Forsythe): Failed (P < 0.050)	Friedman Repeated Measures Analysis of Variance on Ranks		Main effect of time	<0.001	χ2 = 41.782 with 9 degrees of freedom.
					Dunnett's Method	vs. 15 s-averaged baseline	See Fig	

	MAP, after saline	6	Normality Test (Shapiro-Wilk): Passed (P = 0.490) Equal Variance Test (Brown-Forsythe): Failed (P < 0.050)	Friedman Repeated Measures Analysis of Variance on Ranks		Main effect of time	<0.001	χ2 = 47.309 with 9 degrees of freedom.
					Dunnett's Method	vs. 15 s-averaged baseline	See Fig	
	HR, after saline	6	Normality Test (Shapiro-Wilk): Passed (P = 0.668) Equal Variance Test (Brown-Forsythe): Passed (P = 0.673)	One Way Repeated Measures Analysis of Variance		Main effect of time	0.007	F = 2.964 with 9 degrees of freedom.
					Dunnett's Method	vs. 15 s-averaged baseline	See Fig	
	VRNA, after AP5/CNQX	6	Normality Test (Shapiro-Wilk): Passed (P = 0.137) Equal Variance Test (Brown-Forsythe): Failed (P < 0.050)	Friedman Repeated Measures Analysis of Variance on Ranks		Main effect of time	0.257	χ2 = 11.273 with 9 degrees of freedom.

	MAP. after AP5/CNQX	6	Normality Test (Shapiro-Wilk): Passed (P = 0.056) Equal Variance Test (Brown-Forsythe): Passed (P = 0.183)	One Way Repeated Measures Analysis of Variance		Main effect of time	<0.001	F = 24.310 with 9 degrees of freedom.
					Dunnett's Method	vs. 15 s-averaged baseline	See Fig	
	HR, after AP5/CNQX	6	Normality Test (Shapiro-Wilk): Failed (P < 0.050)	Friedman Repeated Measures Analysis of Variance on Ranks		Main effect of time	0.006	χ2 = 23.200 with 9 degrees of freedom.
					Dunnett's Method	vs. 15 s-averaged baseline	See Fig	
Suppl. 4b	distance	8	Normality Test (Shapiro-Wilk): Passed (P = 0.143) Equal Variance Test (Brown-Forsythe): Failed (P < 0.050)	Friedman Repeated Measures Analysis of Variance on Ranks		among frequencies	<0.001	χ2 = 14.250 with 2 degrees of freedom.
					Tukey's	10 vs 20 vs 40	See Fig	

					Method			
	Integrated ΔMAP	8	Normality Test (Shapiro-Wilk): Passed (P = 0.891) Equal Variance Test (Brown-Forsythe): Passed (P = 0.255)	One Way Repeated Measures Analysis of Variance		among frequencies	0.002	F = 10.274 with 2 degrees of freedom.
					Tukey's Method	10 vs 20 vs 40	See Fig	
	Integrated ΔHR	8	Normality Test (Shapiro-Wilk): Passed (P = 0.190) Equal Variance Test (Brown-Forsythe): Passed (P = 0.976)	One Way Repeated Measures Analysis of Variance		among frequencies	0.009	F = 6.760 with 2 degrees of freedom.
					Tukey's Method	10 vs 20 vs 40	See Fig	
	distance	8	Normality Test (Shapiro-Wilk): Passed (P = 0.976) Equal Variance Test (Brown-Forsythe):	One Way Repeated Measures Analysis of Variance		among intensities	0.042	F = 3.998 with 2 degrees of freedom.

			Passed (P = 0.597)					
					Tukey's Method	10 vs 20 vs 35~40	See Fig	
	Integrated ΔMAP	8	Normality Test (Shapiro-Wilk): Passed (P = 0.897) Equal Variance Test (Brown-Forsythe): Passed (P = 0.561)	One Way Repeated Measures Analysis of Variance		among intensities	0.01	F = 6.437 with 2 degrees of freedom.
					Tukey's Method	10 vs 20 vs 35~40	See Fig	
	Integrated ΔHR	8	Normality Test (Shapiro-Wilk): Passed (P = 0.655) Equal Variance Test (Brown-Forsythe): Passed (P = 0.402)	One Way Repeated Measures Analysis of Variance		among intensities	0.092	F = 2.836 with 2 degrees of freedom.

Suppl. 4d	distance	8	Normality Test (Shapiro-Wilk): Passed (P = 0.059) Equal Variance Test (Brown-Forsythe): Passed (P = 0.347)	One Way Repeated Measures Analysis of Variance		among frequencies	<0.001	F = 15.974 with 2 degrees of freedom.
					Tukey's Method	10 vs 20 vs 40	See Fig	
	Integrated ΔMAP	8	Normality Test (Shapiro-Wilk): Passed (P = 0.785) Equal Variance Test (Brown-Forsythe): Passed (P = 0.060)	One Way Repeated Measures Analysis of Variance		among frequencies	<0.001	F = 23.597 with 2 degrees of freedom.
					Tukey's Method	10 vs 20 vs 40	See Fig	
	Integrated ΔHR	8	Normality Test (Shapiro-Wilk): Passed (P = 0.423) Equal Variance Test (Brown-Forsythe): Passed (P = 0.186)	One Way Repeated Measures Analysis of Variance		among frequencies	0.119	F = 2.488 with 2 degrees of freedom.

	distance	8	Normality Test (Shapiro-Wilk): Passed (P = 0.660) Equal Variance Test (Brown-Forsythe): Passed (P = 0.399)	One Way Repeated Measures Analysis of Variance		among intensities	0.48	F = 0.773 with 2 degrees of freedom.
	Integrated ΔMAP	8	Normality Test (Shapiro-Wilk): Passed (P = 0.070) Equal Variance Test (Brown-Forsythe): Passed (P = 0.851)	One Way Repeated Measures Analysis of Variance		among intensities	0.006	F = 7.471 with 2 degrees of freedom.
					Tukey's Method	10 vs 20 vs 35~40	See Fig	
	Integrated ΔHR	8	Normality Test (Shapiro-Wilk): Passed (P = 0.164) Equal Variance Test (Brown-Forsythe): Passed (P = 0.536)	One Way Repeated Measures Analysis of Variance		among intensities	0.544	F = 0.636 with 2 degrees of freedom.

Suppl. 5a	Speed, 35~40 mW, 40 Hz, unilateral	4	Normality Test (Shapiro-Wilk): Passed (P = 0.113) Equal Variance Test (Brown-Forsythe): Passed (P = 0.382)	One Way Repeated Measures Analysis of Variance		Main effect of time	0.168	F = 1.4466 with 15 degrees of freedom.
	MAP, 35~40 mW, 40 Hz, unilateral	4	Normality Test (Shapiro-Wilk): Passed (P = 0.965) Equal Variance Test (Brown-Forsythe): Passed (P = 0.481)	One Way Repeated Measures Analysis of Variance		Main effect of time	0.042	F = 1.957 with 15 degrees of freedom.
					Dunnett's Method	vs. 15 s-averaged baseline	See Fig	
	HR, 35~40 mW, 40 Hz, unilateral	4	Normality Test (Shapiro-Wilk): Passed (P = 0.669) Equal Variance Test (Brown-Forsythe): Passed (P = 0.295)	One Way Repeated Measures Analysis of Variance		Main effect of time	0.103	F = 1.630 with 15 degrees of freedom.

	Speed, 15~20 mW, 40 Hz, bilateral	4	Normality Test (Shapiro-Wilk): Failed (P < 0.050)	Friedman Repeated Measures Analysis of Variance on Ranks		Main effect of time	0.041	$\chi2$ = 25.683 with 15 degrees of freedom.
					Dunnett's Method	vs. 15 s-averaged baseline	See Fig	
	MAP, 15~20 mW, 40 Hz, bilateral	4	Normality Test (Shapiro-Wilk): Passed (P = 0.262) Equal Variance Test (Brown-Forsythe): Passed (P = 0.959)	One Way Repeated Measures Analysis of Variance		Main effect of time	0.991	F = 0.318 with 15 degrees of freedom.
	HR, 15~20 mW, 40 Hz, bilateral	4	Normality Test (Shapiro-Wilk): Passed (P = 0.357) Equal Variance Test (Brown-Forsythe): Passed (P = 0.373)	One Way Repeated Measures Analysis of Variance		Main effect of time	0.006	F = 2.663 with 15 degrees of freedom.
					Dunnett's Method	vs. 15 s-averaged baseline	See Fig	

Suppl. 5b	Speed, 35~40 mW, 40 Hz, unilateral	4	Normality Test (Shapiro-Wilk): Passed (P = 0.648) Equal Variance Test (Brown-Forsythe): Passed (P = 0.062)	One Way Repeated Measures Analysis of Variance		Main effect of time	0.054	F = 1.867 with 15 degrees of freedom.
	MAP, 35~40 mW, 40 Hz, unilateral	4	Normality Test (Shapiro-Wilk): Failed (P < 0.050)	Friedman Repeated Measures Analysis of Variance on Ranks		Main effect of time	0.434	$\chi2$ = 15.243 with 15 degrees of freedom.
	HR, 35~40 mW, 40 Hz, unilateral	4	Normality Test (Shapiro-Wilk): Passed (P = 0.557) Equal Variance Test (Brown-Forsythe): Passed (P = 0.652)	One Way Repeated Measures Analysis of Variance		Main effect of time	0.939	F = 0.480 with 15 degrees of freedom.
	Speed, 15~20 mW, 40 Hz, bilateral	4	Normality Test (Shapiro-Wilk): Failed (P < 0.050)	Friedman Repeated Measures Analysis of Varianc		Main effect of time	0.147	$\chi2$ = 20.682 with 15 degrees of freedom.

				e on Ranks				
	MAP, 15~20 mW, 40 Hz, bilateral	4	Normality Test (Shapiro-Wilk): Passed (P = 0.367) Equal Variance Test (Brown-Forsythe): Passed (P = 0.365)	One Way Repeated Measures Analysis of Variance		Main effect of time	0.056	F = 1.852 with 15 degrees of freedom.
	HR, 15~20 mW, 40 Hz, bilateral	4	Normality Test (Shapiro-Wilk): Passed (P = 0.198) Equal Variance Test (Brown-Forsythe): Passed (P = 0.982)	One Way Repeated Measures Analysis of Variance		Main effect of time	0.945	F = 0.467 with 15 degrees of freedom.
Suppl. 6c	speed	5	Normality Test (Shapiro-Wilk): Failed (P < 0.050)	Friedman Repeated Measures Analysis of Variance on Ranks		Main effect of time	0.074	$\chi2$ = 23.510 with 15 degrees of freedom.

	MAP	5	Normality Test (Shapiro-Wilk): Passed (P = 0.680) Equal Variance Test (Brown-Forsythe): Passed (P = 0.084)	One Way Repeated Measures Analysis of Variance		Main effect of time	<0.001	F = 3.735 with 15 degrees of freedom.
	HR	5	Normality Test (Shapiro-Wilk): Passed (P = 0.509) Equal Variance Test (Brown-Forsythe): Passed (P = 0.980)	One Way Repeated Measures Analysis of Variance		Main effect of time	0.472	F = 0.995 with 15 degrees of freedom.
Suppl. 7d	Mean percentrage of Fos expression per GFP-positive population	12, 4/group	Normality Test (Shapiro-Wilk): Passed (P = 0.888)<br><br>Equal Variance Test (Brown-Forsythe): Passed (P = 0.116)	One Way Analysis of Variance		Main effect of rat group	<0.001	F = 36.766 with 11 degrees of freedom.
					Tukey Test	ChR2 vs. ctl	<0.001	
					Tukey Test	ChR2 vs. ChR2&iChloC	<0.001	

					Tukey Test	ctl vs. ChR2&iChloC	0.180	
Suppl. 8b	trial percentages	4, control; 7, iChloC	Normality Test (Shapiro-Wilk): Passed (P = 0.342)<br><br>Equal Variance Test (Brown-Forsythe): Passed (P = 0.163)	Two Way Repeated Measures ANOVA (One Factor Repetition)		control vs. iChloC, among behavioral patterns	behavioral patterns, <0.001; rat group X behavioral patterns, <0.001	F = 14.456 (behavioral patterns), F = 21.969 (rat group X behavioral patterns)
					Tukey's Method	all pairs	See Fig	
Suppl. 9	MAP, control	4	Normality Test (Shapiro-Wilk): Passed (P = 0.695) Equal Variance Test (Brown-Forsythe): Passed (P = 0.422)	One Way Repeated Measures Analysis of Variance		Main effect of time	0.355	F = 1.073 with 60 degrees of freedom.
	HR, control	4	Normality Test (Shapiro-Wilk): Passed (P = 0.352) Equal Variance Test (Brown-Forsythe): Passed (P = 0.634)	One Way Repeated Measures Analysis of Variance		Main effect of time	<0.001	F =7.400 with 60 degrees of freedom.

					Holm-Sidak's Method	vs. 2 s-averaged baseline	See Fig	
	MAP, iChloC	7	Normality Test (Shapiro-Wilk): Failed (P < 0.050)	Friedman Repeated Measures Analysis of Variance on Ranks		Main effect of time	0.258	$\chi 2 = 67.486$ with 60 degrees of freedom.
	HR, iChloC	7	Normality Test (Shapiro-Wilk): Passed (P = 0.802) Equal Variance Test (Brown-Forsythe): Passed (P = 0.670)	One Way Repeated Measures Analysis of Variance		Main effect of time	<0.001	F =1.982 with 60 degrees of freedom.
					Holm-Sidak’s Method	vs. 2 s-averaged baseline	See Fig	
