# Supplementary material for: A brainstem monosynaptic excitatory pathway that drives locomotor activities and sympathetic cardiovascular responses
Source: Nat Commun. 2022 Aug 29;13:5079. doi: 10.1038/s41467-022-32823-x (PMC9424289; doi:10.1038/s41467-022-32823-x)
Supplement: Supplementary file 3 — Description of Additional Supplementary Files [file 41467_2022_32823_MOESM3_ESM.pdf]

File name: Supplementary Movie 1

Description: Overhead view of an experiment in which a ChR2-GFP-expressing conscious rat left free in the circular track received 15-s sustained optogenetic excitatory intervention at 40 Hz (20 mW). Locomotor speed and cardiovascular changes during this recording are presented in Fig. 4d.

File name: Supplementary Movie 2

Description: Overhead view of an experiment in which a ChR2-GFP-expressing conscious rat left free in the circular track received five-times-repeated optogenetic excitatory interventions (5-s laser-on/5-s off) at 40 Hz (20 mW). Locomotor speed and cardiovascular changes during this recording are presented in Fig. 4f.

File name: Supplementary Movie 3

Description: Overhead view of an experiment in which an iChloC-mcherryexpressing conscious rat received 2-s optogenetic inhibitory intervention during voluntary running on the wheel. Wheel rotation ratio and cardiovascular changes during this recording are shown in Fig. 5d.
