# Supplementary material for: A brainstem monosynaptic excitatory pathway that drives locomotor activities and sympathetic cardiovascular responses
Source: Nat Commun. 2022 Aug 29;13:5079. doi: 10.1038/s41467-022-32823-x (PMC9424289; doi:10.1038/s41467-022-32823-x)
Supplement: Supplementary file 7 — Reporting Summary [file 41467_2022_32823_MOESM7_ESM.pdf]

nature portfolio

Corresponding author(s): Satoshi Koba

Last updated by author(s): Aug 8, 2022

Reporting Summary

Nature Portfolio wishes to improve the reproducibility of the work that we publish. This form provides structure for consistency and transparency in reporting. For further information on Nature Portfolio policies, see our Editorial Policies and the Editorial Policy Checklist.

Statistics

For all statistical analyses, confirm that the following items are present in the figure legend, table legend, main text, or Methods section.

n/a	Confirmed	
☐	☒	The exact sample size (n) for each experimental group/condition, given as a discrete number and unit of measurement
☐	☒	A statement on whether measurements were taken from distinct samples or whether the same sample was measured repeatedly
☐	☒	The statistical test(s) used AND whether they are one- or two-sided<br>*Only common tests should be described solely by name; describe more complex techniques in the Methods section.*
☒	☐	A description of all covariates tested
☐	☒	A description of any assumptions or corrections, such as tests of normality and adjustment for multiple comparisons
☐	☒	A full description of the statistical parameters including central tendency (e.g. means) or other basic estimates (e.g. regression coefficient) AND variation (e.g. standard deviation) or associated estimates of uncertainty (e.g. confidence intervals)
☐	☒	For null hypothesis testing, the test statistic (e.g. F, t, r) with confidence intervals, effect sizes, degrees of freedom and P value noted<br>*Give P values as exact values whenever suitable.*
☒	☐	For Bayesian analysis, information on the choice of priors and Markov chain Monte Carlo settings
☒	☐	For hierarchical and complex designs, identification of the appropriate level for tests and full reporting of outcomes
☒	☐	Estimates of effect sizes (e.g. Cohen's d, Pearson's r), indicating how they were calculated

Our web collection on statistics for biologists contains articles on many of the points above.

Software and code

Policy information about availability of computer code

Data collection	PowerLab 8/30 and 8/35, LabChart version 8.0 or 8.1.16 (ADInstruments); Smart 3.0 (Panlab); TR181 SmartPad, TR190 Configurator, TRM54P Pressure Telemeter (Millar/Kaha Sciences); LSM780 confocal microscope, ZEN 2.3 (Carl Zeiss); BZ-9000 and BZ-X710 all-in-one fluorescence microscope (Keyence)
Data analysis	Borland C++ 10.2 (Borland); CorelDRAW X6 (Corel); KaleidaGraph 4.4.5 (Synergy Software); Sigmaplot 14.0 (Systat); Excel (Office)

For manuscripts utilizing custom algorithms or software that are central to the research but not yet described in published literature, software must be made available to editors and reviewers. We strongly encourage code deposition in a community repository (e.g. GitHub). See the Nature Portfolio guidelines for submitting code & software for further information.

Data

Policy information about availability of data

All manuscripts must include a data availability statement. This statement should provide the following information, where applicable:

- Accession codes, unique identifiers, or web links for publicly available datasets
- A description of any restrictions on data availability
- For clinical datasets or third party data, please ensure that the statement adheres to our policy

All data associated with this study are provided in this published article and its supplementary information files. Source data are provided with this paper.

Human research participants

Policy information about studies involving human research participants and Sex and Gender in Research.

Reporting on sex and gender	NA
Population characteristics	NA
Recruitment	NA
Ethics oversight	NA

Note that full information on the approval of the study protocol must also be provided in the manuscript.

Field-specific reporting

Please select the one below that is the best fit for your research. If you are not sure, read the appropriate sections before making your selection.

☒ Life sciences ☐ Behavioural & social sciences ☐ Ecological, evolutionary & environmental sciences

For a reference copy of the document with all sections, see nature.com/documents/nr-reporting-summary-flat.pdf

Life sciences study design

All studies must disclose on these points even when the disclosure is negative.

Sample size	The sample size was not determined with statistical methods but chosen based on previous previous experience and standards in this field (e.g., 10.1113/JP276813, 10.1126/science.aaz4639). Sample sizes were described in the manuscript.
Data exclusions	Data were excluded from results in cases that any injection missed the targeted region or that the tips of optic fibers was inadequately located. In the experiments to test the effect of optogenetic stimulation in conscious animals, data were discarded if disconnection of telemetry signals more than 1 s occurred during optogenetic interventions. In the experiments to test the effect of optogenetic inhibition, data were not collected on rats which were not willing to voluntarily run on the wheel.
Replication	Experiments were performed with sufficient animals per group to demonstrate statistical significance. In experiments of Figure 2a-d, 3a-d, 4, Supplementary Fig 2, 3a, 3c-d, 4, 5, and 6, attempts at replication (2-4 times in each rat) were successful. Other experiments were performed independently.
Randomization	Littermates were randomly assigned into control or different experimental groups.
Blinding	Assistants who were well trained but were not informed of the animal identification performed manual cell counting (Figure 1j-k, Supplementary Fig 1c-d, Supplementary Fig 7). In other analyses, blind was not performed because data acquisitions and analysis procedures were automated and have a high objectivity.

Reporting for specific materials, systems and methods

We require information from authors about some types of materials, experimental systems and methods used in many studies. Here, indicate whether each material, system or method listed is relevant to your study. If you are not sure if a list item applies to your research, read the appropriate section before selecting a response.

Materials & experimental systems

n/a	Involved in the study	
☐	☒	Antibodies
☐	☒	Eukaryotic cell lines
☒	☐	Palaeontology and archaeology
☐	☒	Animals and other organisms
☒	☐	Clinical data
☒	☐	Dual use research of concern

Methods

n/a	Involved in the study	
☒	☐	ChIP-seq
☒	☐	Flow cytometry
☒	☐	MRI-based neuroimaging

Antibodies

Antibodies used	Commercially available antibodies were used.<br>Primary antibodies:<br>chicken anti-tyrosine hydroxylase (1:500, Abcam, ab76442, GR3190915-1), goat anti-choline acetyltransferase (1:100~1:200, Merck, AB144P, 2843047, 3315729), goat-anti GFP (1:1000, GeneTex, GTX26673, 82160686), goat anti-tdTomato (1:500, Sicgen, AB8181, ACR-2324-72), mouse anti-Cre Recombinase (1:1000, Merck, MAB3120, 2987468), rabbit anti-c-Fos (1:400, Cell Signaling Technology, 2250s, P01100), rabbit anti-GFP (1:1000, Invitrogen, A-6455, 244346), rabbit anti-RFP (1:1000, Rockland, 600-401-379, 35411), rabbit anti-tyrosine hydroxylase (1:1000, Merck, AB152, 2745367), and rabbit anti-vesicular glutamate transporter 2 (1:500, Frontier Institute, AF860).<br>Secondary antibodies:<br>donkey anti-chicken DyLight 405 (1:250, Jackson ImmunoResearch, 703-475-155, 130733), donkey anti-chicken Alexa Fluor 488 (1:500, Jackson ImmunoResearch, 703-545-155, 132771), donkey anti-goat Alexa Fluor 405 (1:500, Abcam, ab175665, GR3242721-2), donkey anti-goat Alexa Fluor 488 (1:500, Abcam, ab150129, GR3351032-1), donkey anti-goat Alexa Fluor 555 (1:500, Thermo fisher Scientific, A21432, or Abcam, ab150130, GR3220578-2), donkey anti-mouse Alexa Fluor 488 (1:500, Abcam, ab150109, GR3296119-1), donkey anti-mouse Alexa Fluor 555 (1:500, Abcam, ab150106, GR3220544-1), donkey anti-rabbit Alexa Fluor 488 (1:500, Thermo fisher Scientific, A-21206, or Abcam, ab150073, GR226381-3), and donkey anti-rabbit Alexa Fluor 555 (1:500, Abcam, ab150074, GR3241278-1).
Validation	All antibodies have been previously published and were validated as stated on the manufacturer's websites. Negative control experiments without primary antibodies showed no fluorescence. Validation statements of primary antibodies and previous publications can be found as following.<br>chicken anti-tyrosine hydroxylase (Abcam, ab76442): https://www.abcam.com/tyrosine-hydroxylase-antibody-ab76442.html, 10.1126/sciadv.aaz4232<br>goat anti-choline acetyltransferase (Merck, AB144P): https://www.merckmillipore.com/JP/ja/product/Anti-Choline-Acetyltransferase-Antibody,MM_NF-AB144P?ReferrerURL=https%3A%2F%2Fwww.google.com%2F, 10.1038/s41467-020-16053-7<br>goat-anti GFP (GeneTex, GTX26673): https://www.genetex.com/Product/Detail/GFP-antibody/GTX26673, 10.1016/j.neuron.2014.09.023<br>goat anti-tdTomato (Sicgen, AB8181): http://www.sicgen.pt/product/tdtomato-polyclonal-antibody_1_135, 10.1038/s41598-020-76870-0<br>mouse anti-Cre Recombinase (1:1000, Merck, MAB3120): https://www.merckmillipore.com/JP/ja/product/Anti-Cre-Recombinase-Antibody-clone-2D8,MM_NF-MAB3120, 10.1038/s41598-018-23810-8<br>rabbit anti-c-Fos (Cell Signaling Technology, 2250s): https://www.cellsignal.jp/products/primary-antibodies/c-fos-9f6-rabbit-mab/2250, 10.1016/j.cell.2017.06.045<br>rabbit anti-GFP (1:1000, Invitrogen, A-6455): https://www.thermofisher.com/antibody/product/GFP-Antibody-Polyclonal/A-6455, 0.1038/s41467-019-11092-1<br>rabbit anti-RFP (1:1000, Rockland, 600-401-379): https://rockland-inc.com/store/Antibodies-to-GFP-and-Antibodies-to-RFP-600-401-379-O4L_24299.aspx, 10.1016/j.cell.2015.10.074<br>rabbit anti-tyrosine hydroxylase (Merck, AB152): https://www.merckmillipore.com/JP/ja/product/Anti-Tyrosine-Hydroxylase-Antibody,MM_NF-AB152?ReferrerURL=https%3A%2F%2Fwww.google.com%2F, 10.1523/JNEUROSCI.0195-15.2015<br>rabbit anti-vesicular glutamate transporter 2 (Frontier Institute, AF860): https://nittobo-nmd.co.jp/pdf/reagents/VGluT2.pdf, 10.1002/jnr.24214

Eukaryotic cell lines

Policy information about cell lines and Sex and Gender in Research

Cell line source(s)	HEK293T cells were obtained from RIKEN BRC Cell Bank (RBRC-RCB2202).
Authentication	HEK293T cells were authenticated by the RIKEN BRC Cell Bank.
Mycoplasma contamination	Cell line was regularly tested for mycoplasma contamination by PCR.
Commonly misidentified lines (See ICLAC register)	N/A

Animals and other research organisms

Policy information about studies involving animals; ARRIVE guidelines recommended for reporting animal research, and Sex and Gender in Research

Laboratory animals	Sprague Dawley rats (Slc:SD, male and female, 8~10 weeks) were purchased from Shimizu Laboratory Supplier Co, Ltd (Kyoto, Japan) originally from Japan SLC, Inc (Hamamatsu, Japan), and bred in our facilities. All rats were maintained in a room air-conditioned at 25 ℃ with a 12 h:12 h light:dark cycle. Rats were housed in standard cages besides rats used in experiments to study the effect of optogenetic inhibition, which were housed in cages with flying saucer wheels after weaning. Food and water were made available ad libitum.
Wild animals	No wild animal was used in this study.
Reporting on sex	Sex of the animals is reported in each figure legend and sex of each animal is reported in the source data. Totally 107 male and 9 female rats were used.

Field-collected samples	No field-collected sample was used in this study.
Ethics oversight	All procedures were approved by the Animal Care Committee (ref#: 15-Y-40, 18-Y-11, 19-Y-53) and the Gene Recombination Experiment Safety Committee (ref#: 28-034, 31-067, 32-061) of Tottori University.

Note that full information on the approval of the study protocol must also be provided in the manuscript.
